# Pirated Siderophores Promote Sporulation in Bacillus subtilis

**DOI:** 10.1128/AEM.03293-16

**Published:** 2017-05-01

**Authors:** Gabrielle M. Grandchamp, Lews Caro, Elizabeth A. Shank

**Affiliations:** a Department of Biology, University of North Carolina at Chapel Hill, Chapel Hill, North Carolina, USA; b Department of Microbiology and Immunology, University of North Carolina at Chapel Hill, Chapel Hill, North Carolina, USA; c Curriculum of Genetics and Molecular Biology, University of North Carolina at Chapel Hill, Chapel Hill, North Carolina, USA; University of Georgia

**Keywords:** Bacillus subtilis, Escherichia coli, cell-cell interaction, coculture, secondary metabolites, siderophores, specialized metabolites, sporulation

## Abstract

In microbial communities, bacteria chemically and physically interact with one another. Some of these interactions are mediated by secreted specialized metabolites that act as either intraspecies or interspecies signals to alter gene expression and to change cell physiology. Bacillus subtilis is a well-characterized soil microbe that can differentiate into multiple cell types, including metabolically dormant endospores. We were interested in identifying microbial interactions that affected sporulation in B. subtilis. Using a fluorescent transcriptional reporter, we observed that coculturing B. subtilis with Escherichia coli promoted sporulation gene expression via a secreted metabolite. To identify the active compound, we screened the E. coli Keio Collection and identified the sporulation-accelerating cue as the siderophore enterobactin. B. subtilis has multiple iron acquisition systems that are used to take up the B. subtilis-produced siderophore bacillibactin, as well as to pirate exogenous siderophores such as enterobactin. While B. subtilis uses a single substrate binding protein (FeuA) to take up both bacillibactin and enterobactin, we discovered that it requires two distinct genes to sporulate in response to these siderophores (the esterase gene *besA* for bacillibactin and a putative esterase gene, *ybbA*, for enterobactin). In addition, we found that siderophores from a variety of other microbial species also promote sporulation in B. subtilis. Our results thus demonstrate that siderophores can act not only as bacterial iron acquisition systems but also as interspecies cues that alter cellular development and accelerate sporulation in B. subtilis.

**IMPORTANCE** While much is known about the genetic regulation of Bacillus subtilis sporulation, little is understood about how other bacteria influence this process. This work describes an interaction between Escherichia coli and B. subtilis that accelerates sporulation in B. subtilis. The interaction is mediated by the E. coli siderophore enterobactin; we show that other species' siderophores also promote sporulation gene expression in B. subtilis. These results suggest that siderophores not only may supply bacteria with the mineral nutrient iron but also may play a role in bacterial interspecies signaling, providing a cue for sporulation. Siderophores are produced by many bacterial species and thus potentially play important roles in altering bacterial cell physiology in diverse environments.

## INTRODUCTION

Bacteria live in communities where they have the potential to interact with different microbial species. Many intraspecies and interspecies bacterial interactions are mediated by secreted signaling molecules, some of which alter gene expression and affect cellular physiology (1–8). The activities of many specialized metabolites remain largely uncharacterized, however, and such metabolites may have unexpected roles in microbial communities (3, 9).

The bacterium Bacillus subtilis is a common soil microbe that is an excellent organism in which to study cellular differentiation. B. subtilis differentiates into multiple distinct cell types, including endospores (here simply called spores) (10). Spores are metabolically dormant cell bodies that are highly resistant to heat, UV radiation, and antibiotics. Traditionally, B. subtilis has been thought to sporulate when exposed to harsh environmental conditions (11–13). The decision to sporulate is a tightly controlled process that involves a complex regulatory network that is sensitive to environmental conditions (10, 13–16). However, many of the specific environmental cues that lead to sporulation remain unknown (10, 13, 14).

Although multiple bacterial metabolites alter B. subtilis cellular physiology (5, 7, 8), the only specialized metabolite previously shown to have a specific effect on sporulation is decoyinine (an inhibitor of GMP synthesis produced by Streptomyces hygroscopicus) (17, 18). We predicted that other microbial metabolites might also have the ability to alter B. subtilis sporulation. To identify such metabolites, we used a B. subtilis strain containing a fluorescent transcriptional reporter in which the promoter for *sspB* (P_*sspB*_), a gene encoding a protein that is highly upregulated during the committed stage of sporulation (19, 20), drives expression of the gene encoding the yellow fluorescent protein (*yfp*). This entire construct is inserted into the neutral locus *amyE* in the B. subtilis genome (21). In this strain (*amyE*::P_*sspB*_-*yfp*), fluorescence provides a measure of sporulation promoter activity, which acts as a proxy for expression of sporulation genes. We used this reporter strain in a coculture assay and determined that Escherichia coli promotes B. subtilis sporulation gene expression. Using an E. coli knockout library, we further determined that this interaction is dependent on production of the siderophore enterobactin.

Most bacteria require iron to survive. However, bacteria living in the soil encounter concentrations of soluble iron that are much lower than those required for survival (22–24). As a consequence of this poor iron bioavailability, bacteria secrete siderophores (low-molecular-weight compounds that exhibit high binding affinity for iron) as iron-scavenging mechanisms (22, 24, 25). Bacteria possess binding proteins and transporters to take up specific iron-siderophore complexes, as well as mechanisms to release the iron from the siderophores inside the cells (22). Because siderophores are secreted as a “public good,” siderophore piracy (in which bacteria possess the uptake machinery for siderophores produced by other microbes) is common (26–32). Indeed, some bacteria appear to rely on siderophore piracy for survival; many “uncultivable” bacteria grow when provided with siderophores from other bacteria (33).

B. subtilis and E. coli both synthesize their own tricatecholate siderophores, i.e., bacillibactin and enterobactin, respectively (34–38). The production and transport of these siderophores are Fur regulated (39–42). In addition to making bacillibactin, B. subtilis has the ability to pirate a range of xenosiderophores, including enterobactin (28, 31, 32). The B. subtilis substrate binding protein (SBP) FeuA is able to bind both bacillibactin and enterobactin (35, 43–45). The esterase BesA, which releases iron from bacillibactin inside the cells, is able to hydrolyze enterobactin *in vitro* (43), albeit less efficiently than it hydrolyzes bacillibactin (45).

In this work, we show that, in addition to its known role as an iron chelator, purified enterobactin acts as an interspecies modulator of cellular physiology, increasing both sporulation gene expression and the timing of viable spore formation in B. subtilis. We demonstrate that this effect on B. subtilis sporulation is not specific to enterobactin but also occurs in response to other purified siderophores. Therefore, our results support an important role for siderophores in stimulating sporulation in B. subtilis, and they suggest that the availability and diversity of siderophores present in the environment may affect not only survival (by determining bacterial access to iron), but also sporulation of some members of the community.

## RESULTS

### Escherichia coli promotes sporulation gene expression in Bacillus subtilis.

Using a modification of an established coculture screen (7, 46), we used the B. subtilis P_*sspB*_-*yfp* reporter strain to identify bacterial species that stimulated sporulation gene expression in B. subtilis (Table 1). In short, we grew B. subtilis P_*sspB*_-*yfp* as lawns of microcolonies on 0.1× Luria-Bertani (LB) medium-100 mM MOPS [3-(*N*-morpholino)propanesulfonic acid] (pH 7) agar plates (∼25,000 colonies per 10-cm-diameter plate). The 0.1× LB-Lennox agar medium was selected for our assays because it is relatively nutrient poor (similar to the oligotrophic environment of soil) but is still able to support the growth of diverse microbes; it was preferred over the medium typically used to investigate sporulation, i.e., Difco sporulation medium (DSM), because spores form too rapidly in DSM, limiting our ability to identify microbial promotion of sporulation. Cell suspensions of other bacterial species were spotted on the B. subtilis microcolony lawns, which were then assayed for fluorescence. Our unpublished data from a screen of soil microbes indicated that an environmental isolate from the Enterobacteriaceae family promoted sporulation gene expression in B. subtilis. Because our previously published study indicated that closely related bacterial strains frequently have similar effects on B. subtilis cellular differentiation (7, 8), we decided to test the ability of a genetically tractable Enterobacteriaceae, E. coli, to promote sporulation gene expression in B. subtilis.

**TABLE 1:** Strains used in this study

Strain name	Bacterial species/strain	Genotype	Source
NCBI3610	B. subtilis 3610	Wild type	Shank lab collection
ES66	B. subtilis 3610	*amyE*::P*_sspB_*-*yfp*	Shank lab collection
BKE01630	B. subtilis 168	*trpC2 feuA::erm*	BGSC^*a*^
BKE03830	B. subtilis 168	*trpC2 yclQ::erm*	BGSC
BKE07520	B. subtilis 168	*trpC2 yfmC::erm*	BGSC
BKE08440	B. subtilis 168	*trpC2 yfiY::erm*	BGSC
BKE32000	B. subtilis 168	*trpC2 dhbA::erm*	BGSC
BKE32010	B. subtilis 168	*trpC2 besA::erm*	BGSC
BKE33320	B. subtilis 168	*trpC2 fhuD::erm*	BGSC
BKE39610	B. subtilis 168	*trpC2 yxeB::erm*	BGSC
HB8240	B. subtilis 168	*trpC2 att SP*β *sfp^+^ ybbA*::*kan*	John Helmann
ES607	B. subtilis 3610	*kinB*::*erm*	BGSC
ES809	B. subtilis 3610	*amyE*::P*_sspB_*-*yfp feuA*::*erm*	This study
ES825	B. subtilis 3610	*amyE*::P*_sspB_*-*yfp fpiA*::*erm*	This study
ES826	B. subtilis 3610	*amyE*::P*_sspB_*-*yfp yfmC*::*erm*	This study
ES827	B. subtilis 3610	*amyE*::P*_sspB_*-*yfp yfiY*::*erm*	This study
ES810	B. subtilis 3610	*amyE*::P*_sspB_*-*yfp dhbA*::*erm*	This study
ES828	B. subtilis 3610	*amyE*::P*_sspB_*-*yfp fhuD*::*erm*	This study
ES829	B. subtilis 3610	*amyE*::P*_sspB_*-*yfp yxeB*::*erm*	This study
ES811	B. subtilis 3610	*amyE*::P*_sspB_*-*yfp besA*::*erm*	This study
ES812	B. subtilis 3610	*amyE*::P*_sspB_*-*yfp ybbA*::*kan*	This study
ES813	B. subtilis 3610	*amyE*::P*_sspB_*-*yfp besA*::*erm ybbA*::*kan*	This study
ES814	B. subtilis 3610	*amyE*::P*_sspB_*-*yfp dhbA*::*erm ybbA*::*kan*	This study
ES836	B. subtilis 3610	*amyE*::P*_sspB_*-*yfp kinB*::*mls*	This study
ES890	B. subtilis 3610	*lacA*::P_*xyl*_-*ybbA amyE*::P_*sspB*_-*yfp ybbA*::*kan*	This study
ES69	E. coli BW25113	Wild type	Shank lab collection
JW0578	E. coli BW25113	*entF*::*kan*	Keio Collection, from Meta Kuehn
JW0585	E. coli BW25113	*entC*::*kan*	Keio Collection, from Meta Kuehn
JW0586	E. coli BW25113	*entE*::*kan*	Keio Collection, from Meta Kuehn
JW0587	E. coli BW25113	*entB*::*kan*	Keio Collection, from Meta Kuehn
JW0588	E. coli BW25113	*entA*::*kan*	Keio Collection, from Meta Kuehn
JW3440	E. coli BW25113	*acpT*::*kan*	Keio Collection, from Meta Kuehn
ATCC 4513	*B. circulans*	Wild type	Shank lab collection

a BGSC, Bacillus Genetic Stock Center.

**FIG 1:**
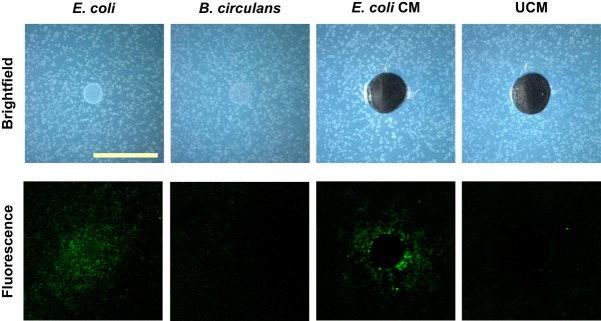
E. coli promotes sporulation gene expression of B. subtilis in coculture, and the sporulation-inducing compound is secreted. Images of microcolony lawns of B. subtilis P_*sspB*_-*yfp* plated on agar plates either with cell suspensions of E. coli (first column) or Bacillus circulans (second column) or with wells filled with E. coli conditioned medium (CM) (third column) or unconditioned medium (UCM) (fourth column) are shown. Bright-field representative images (top row) demonstrate the configuration of the assay (scale bar = 1 cm). Representative fluorescence images (bottom row) demonstrate fluorescent B. subtilis colonies (false-colored green) around the E. coli colony and the E. coli conditioned medium but not around the negative-control colony (*B. circulans*) or the unconditioned medium.

When E. coli was spotted onto lawns of B. subtilis P_*sspB*_-yfp, we saw fluorescence of the B. subtilis microcolonies surrounding the E. coli colony after approximately 25 h (Fig. 1); this signifies that E. coli is promoting sporulation gene expression in B. subtilis either by secreting a signaling compound or by depleting a component of the growth medium. To distinguish between these possibilities, we used conditioned medium (cell-free spent medium) from E. coli to demonstrate that the sporulation-promoting activity was secreted (Fig. 1). We did not see an increase in fluorescence when either a control colony or unconditioned medium was added to the B. subtilis P_*sspB*_-*yfp* microcolony lawns (Fig. 1).

### E. coli mutants that do not accelerate sporulation were identified.

We then wanted to identify the molecule that E. coli was secreting that promoted sporulation gene expression in B. subtilis. To do so, we used the Keio Collection, which contains 3,985 viable single-gene deletion mutants of E. coli strain K-12 BW25113 (47). Using the Keio Collection allowed us to screen for E. coli mutants that no longer promoted fluorescence in B. subtilis P_*sspB*_-*yfp*, providing information about genes that are important for producing the sporulation-promoting factor(s). The Keio Collection strains were grown in shaking liquid culture in 96-well plates for approximately 18 h at 37°C. To confirm growth of the mutants, readings of the optical density at 600 nm (OD_600_) were obtained for all strains. Aliquots of the cultures of each viable mutant were then spotted onto microcolony lawns of B. subtilis P_*sspB*_-*yfp*. Of the 3,985 mutants screened, 115 did not stimulate sporulation and 110 showed increased sporulation stimulation (see Table S1 in the supplemental material). Mutants that no longer promoted sporulation were mapped to the metabolic pathways of E. coli using EcoCyc (48). We were interested in hits within metabolic pathways that involved secreted metabolites. Our EcoCyc analysis indicated that we had multiple hits in the enterobactin biosynthesis pathway. Other mutants that no longer promoted sporulation were involved in the biosynthesis of enterobactin precursors and cell transporters. One of the hits that led to increased sporulation levels was a mutant of the protein required to take up iron-bound enterobactin (FepC); a mutation in FepC would potentially lead to the accumulation of enterobactin outside the E. coli cells, leading to higher levels of the siderophore being available for B. subtilis to pirate. Many of the remaining hits (both positive and negative) involved genes involved in general metabolism and cellular transport. In sum, these data implicated enterobactin as a putative sporulation-promoting factor secreted by E. coli.

### E. coli enterobactin biosynthesis mutants do not accelerate sporulation.

**FIG 2:**
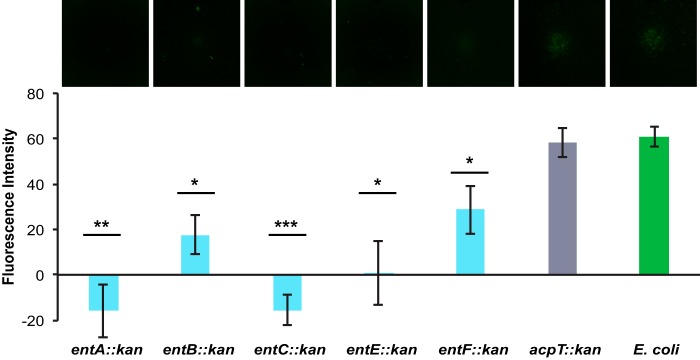
Enterobactin biosynthesis mutants do not promote sporulation gene expression in B. subtilis. The fluorescence intensity of B. subtilis P_*sspB*_-*yfp* microcolony lawns in response to the E. coli strains indicated (*n* = 3) was quantified; a corresponding fluorescence image of each E. coli strain spotted onto the P_*sspB*_-*yfp* microcolony lawn (false-colored green) is shown at the top. The enterobactin biosynthesis mutants (*entA*, *entB*, *entC*, *entE*, and *entF*) showed significantly lower levels of fluorescence (i.e., sporulation gene expression) than did wild-type E. coli or the control acyl carrier protein mutant (*acpT*) strain. Error bars represent standard deviations. *, *P* < 0.05; **, *P* < 0.01; ***, *P* < 0.005 (by *t* test of individual mutants versus wild-type E. coli). Analysis of variance (ANOVA) showed no significant differences among the enterobactin biosynthesis mutants.

Enterobactin is a tricatecholate siderophore (produced by E. coli) that is used to scavenge iron in low-iron environments (37, 49, 50). The biosynthetic pathway for enterobactin requires the actions of nonribosomal peptide synthetases in a multistep enzymatic process (37, 51). To confirm the potential role of enterobactin as a sporulation-accelerating compound, we verified that all of the viable E. coli enterobactin biosynthesis mutants (*entA*, *entB*, *entC*, *entE*, and *entF*) were impaired with respect to sporulation promotion (Fig. 2). The *acpT* mutant was used as a control, since it is not required for enterobactin synthesis but is hypothesized to be involved in the synthesis of related specialized metabolites (Fig. 2).

### Purified enterobactin and high levels of iron accelerate sporulation in B. subtilis.

**FIG 3:**
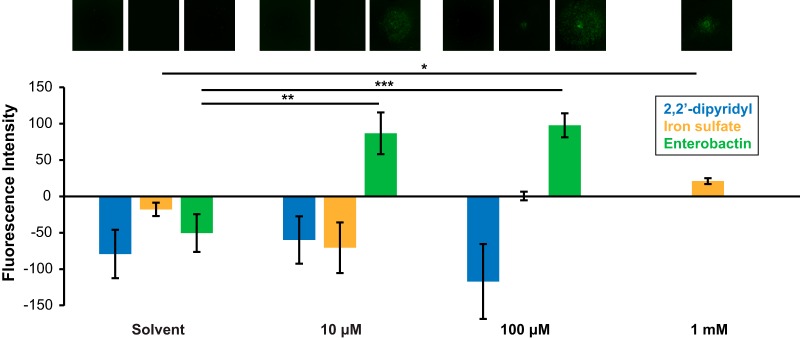
Enterobactin and high levels of iron promote sporulation gene expression. The fluorescence intensity of B. subtilis P_*sspB*_-*yfp* microcolony lawns in response to 2,2′-dipyridyl (blue), iron sulfate (orange), or purified enterobactin (green) at concentrations of 10 μM, 100 μM, or 1 mM (iron sulfate only) or their solvent controls (0 μM) was quantified. The corresponding fluorescence images of the lawns under each condition (false-colored green) are shown at the top. Both enterobactin and iron sulfate significantly stimulated sporulation gene expression, while 2,2′-dipyridyl did not (*n* ≥ 3). Error bars represent standard deviations. *, *P* < 0.05; **, *P* < 0.01; ***, *P* < 0.005.

Enterobactin could potentially promote sporulation gene expression in B. subtilis either by decreasing iron availability (by chelating iron from the medium and rendering it inaccessible to the cells) or by increasing the amount of environmental iron accessible to the cells. To distinguish between these possibilities, B. subtilis sporulation gene expression was assayed in the presence of enterobactin, 2,2′-dipyridyl (a strong iron chelator), and ferrous iron in the form of FeSO_4_. Various concentrations of these compounds were added to wells that had been cored in agar plates, with microcolony lawns of B. subtilis P_*sspB*_-*yfp* plated on top. The addition of 10 μM or 100 μM enterobactin significantly increased sporulation gene expression; 2,2′-dipyridyl had no effect on sporulation gene expression, although we do not know whether, under these conditions, 2,2′-dipyridyl decreased iron levels within the cells (Fig. 3). High concentrations of iron sulfate (∼1 mM) significantly promoted sporulation gene expression, but to a lesser degree than enterobactin (this is likely due to the fact that siderophores allow bacteria to take up iron more efficiently) (Fig. 3). These data indicate that decreased iron availability in the environment does not cause promotion of sporulation gene expression but that increased accessibility to extracellular iron may accelerate this response.

**FIG 4:**
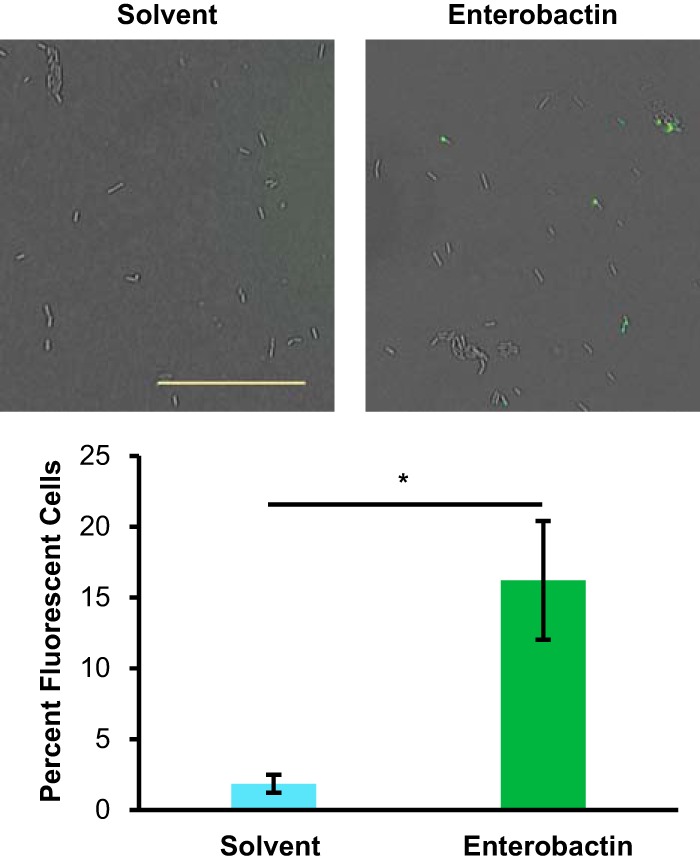
Enterobactin increases the percentage of total fluorescent cells (in which the P_*sspB*_-*yfp* reporter is expressed). The percentage of fluorescent cells was calculated at 24 h for B. subtilis cells treated with 10 μM enterobactin or solvent; corresponding representative fluorescence images overlaid on the bright-field images are shown above (scale bar = 0.25 mm). Both bright-field and fluorescence microscopic images of samples were obtained from microcolony agar assay plates grown in the presence or absence of enterobactin. Total and fluorescent cells were counted to determine the percentage of fluorescent cells in each sample. Samples treated with enterobactin had significantly greater percentages of fluorescent cells than did untreated samples (*n* = 6, with >4,800 cells counted per treatment). Error bars represent standard deviations. *, *P* < 0.01.

Populations of B. subtilis cells often are heterogeneous, with many different cell types (e.g., swimming, biofilm forming, and sporulating) represented within a given population (10, 52). After determining that purified enterobactin promoted sporulation gene expression at the colony level, we wanted to see how enterobactin affected sporulation gene expression at the single-cell level. Enterobactin at 10 μM or a solvent control was added to wells that had been cored in agar plates, and microcolony lawns of B. subtilis P_*sspB*_-*yfp* were plated on top. At 24 h, cells were harvested from around the area of treatment, sheared to break up any clumps, and visualized by microscopy. Bright-field and fluorescence images were obtained to determine the total number of cells and the number of fluorescent cells (in which the P*sspB-yfp* construct was activated), respectively. From these images, we calculated the percentage of fluorescent cells among the enterobactin-treated cells, compared to the control. Enterobactin treatment significantly increased the percentage of fluorescent cells, from approximately 2% to 16% (Fig. 4).

### Enterobactin increases the percentage of viable B. subtilis spores formed.

**FIG 5:**
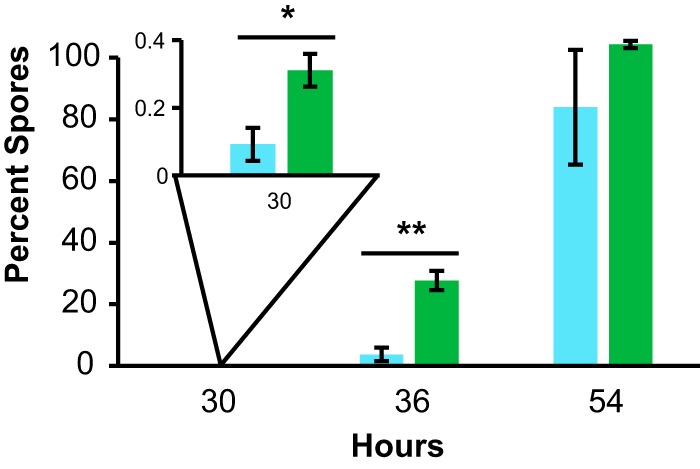
Enterobactin accelerates B. subtilis spore formation. The percentage of spores (of the total number of cells) was calculated at 30, 36, and 54 h for B. subtilis cells treated with 10 μM enterobactin or solvent. Samples were taken from microcolony agar assay plates grown in the presence or absence of enterobactin, and spore counts were performed to determine the percentage of spores in each sample. Samples treated with enterobactin had significantly greater percentages of spores at early time points (30 and 36 h) than did untreated samples (*n* = 3). Error bars represent standard deviations. *, *P* < 0.05; **, *P* < 0.005.

Thus far, we have used the P_*sspB*_-*yfp* fluorescent reporter as a proxy to monitor sporulation gene expression in B. subtilis. To confirm that purified enterobactin also directly affects the formation of viable spores, we performed spore counts. Microcolony lawns of B. subtilis P_*sspB*_-*yfp* were treated with either enterobactin or a solvent control, treated cells were harvested and resuspended, and each sample was divided into two, with one portion being heated at 80°C for 20 min and one portion not receiving heat treatment. Both the unheated and heated aliquots were dilution plated to calculate CFU per milliliter. The unheated samples indicate the total number of viable cells (both vegetative cells and spores), while the heat-treated samples indicate the number of spores present per sample. Cells treated with enterobactin showed significant increases in the percentages of spores formed at 30 and 36 h, compared to untreated cells (Fig. 5), while enterobactin had no effect on overall cell growth (Fig. S1). Thus, enterobactin accelerates the timing of both sporulation gene expression and the formation of viable spores in B. subtilis, without affecting growth.

### Transport of enterobactin into the cells is required for sporulation acceleration.

**FIG 6:**
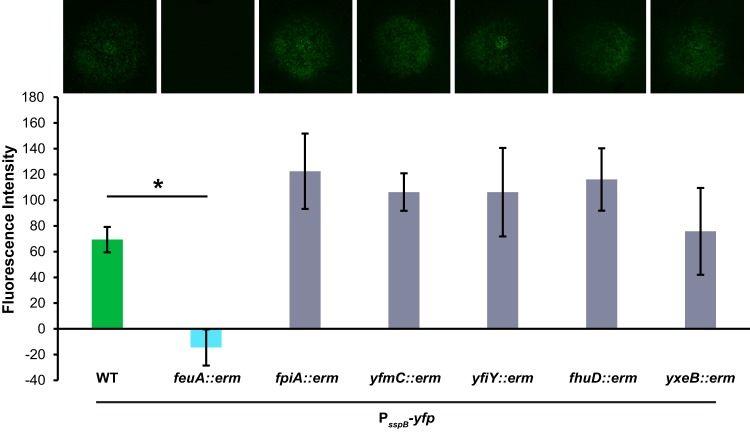
The *feuA* gene is required for enterobactin to promote sporulation gene expression in B. subtilis. The fluorescence intensity of wild-type (WT) or siderophore-specific SBP mutant (*feuA*, *fpiA*, *yfmC*, *yfiY*, *fhuD*, or *yxeB*) B. subtilis P_*sspB*_-*yfp* microcolony lawns in response to 10 μM enterobactin was quantified; corresponding fluorescence images of the lawns (false-colored green) are shown at the top. There was a significant decrease in the promotion of sporulation gene expression on the *feuA* lawns, compared to wild-type B. subtilis (*n* = 3). Error bars represent standard deviations. *, *P* < 0.01.

B. subtilis has six siderophore-specific SBPs (28). These proteins are tethered to the outside of cells, where they bind iron-bound siderophores for uptake into the cells through ABC-type transporters. FeuA is the SBP that binds bacillibactin and enterobactin (35, 43–45). Therefore, we predicted that B. subtilis takes up enterobactin via FeuA and that this uptake is required for sporulation acceleration. To test this prediction, we inserted the sporulation reporter construct (P_*sspB*_-*yfp*) into strains of B. subtilis that lacked each of the siderophore-specific SBPs (*feuA*, *fhuD*, *yxeB*, *fpiA*, *yfmC*, and *yfiY*). Enterobactin was added to agar plates, microcolony lawns of the mutant siderophore-specific SBP reporter strains were added, and fluorescence was monitored. In most of the mutant strains, enterobactin continued to promote fluorescence gene expression in B. subtilis; however, the *feuA* mutant showed no fluorescence activation (Fig. 6). This result indicates that *feuA* is required for enterobactin to promote sporulation gene expression in B. subtilis.

### The *besA* gene is not required for enterobactin to accelerate sporulation in B. subtilis.

**FIG 7:**
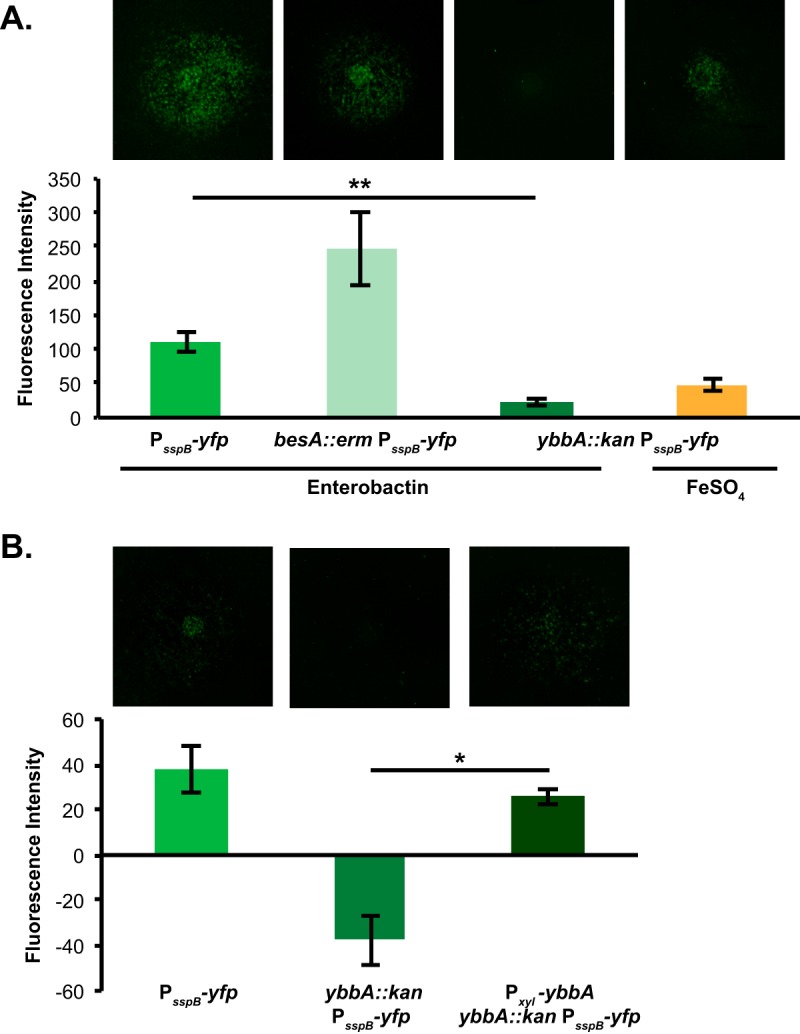
The *ybbA* gene but not *besA* is required for enterobactin to promote sporulation gene expression. (A) The fluorescence intensity of wild-type, *besA*, or *ybbA* B. subtilis P_*sspB*_-*yfp* microcolony lawns in response to 10 μM enterobactin or 1 mM iron sulfate (*ybbA* only) was quantified; corresponding fluorescence images of the lawns (false-colored green) are shown at the top. These data demonstrate that *besA* is not required for enterobactin to promote sporulation gene expression. The promotion of sporulation gene expression was significantly decreased in *ybbA* lawns, compared to wild-type B. subtilis P_*sspB*_-*yfp* lawns, although high levels of iron sulfate could still promote sporulation gene expression in *ybbA* lawns (*n* ≥ 3). (B) The fluorescence intensity of wild-type, *ybbA*, or P_*xyl*_-*ybbA* B. subtilis P_*sspB*_-*yfp* microcolony lawns in response to 10 μM enterobactin on 0.1% xylose was quantified; corresponding fluorescence images of the lawns (false-colored green) are shown at the top. The promotion of sporulation gene expression was significantly decreased in *ybbA* lawns compared to wild-type P_*sspB*_-*yfp* lawns, demonstrating that *ybbA* is required for enterobactin activity (*n* ≥ 3). The ability to respond to enterobactin was restored to wild-type levels in the complemented *ybbA* strain. Error bars represent standard deviations. *, *P* < 0.001; **, *P* < 0.00005.

Once iron-bound siderophores are imported into cells, the iron must be released from these strong chelators in order for the cells to utilize the iron. The esterase BesA is required for B. subtilis to hydrolyze bacillibactin *in vivo*, and BesA is capable of hydrolyzing enterobactin (albeit less efficiently) *in vitro* (45). To determine whether *besA* is required for the sporulation-accelerating activity of enterobactin, we constructed a sporulation reporter with a *besA* mutation (P_*sspB*_-*yfp*, *besA*::*erm*). When enterobactin was added to microcolony lawns of P_*sspB*_-*yfp*, *besA*::*erm*, promotion of sporulation gene expression was observed (Fig. 7A), although the timing was delayed compared to the wild-type strain (fluorescence due to enterobactin was observed at 30 to 32 h in the P_*sspB*_-*yfp*, *besA*::*erm* strain, compared to 24 to 25 h in the wild-type P_*sspB*_-*yfp* strain). This finding suggests that *besA* is not required for enterobactin to promote B. subtilis sporulation. Thus, either the hydrolysis of enterobactin is not required for sporulation acceleration (i.e., the iron-bound siderophore itself may act as a sporulation cue) or B. subtilis has another mechanism to release iron from enterobactin.

### The *ybbA* gene is required for enterobactin to accelerate sporulation in B. subtilis.

Because BesA inefficiently hydrolyzes enterobactin, there has been speculation that B. subtilis encodes another esterase that is better able to hydrolyze enterobactin *in vivo* (45). The *ybbA* gene is a putative esterase whose position in the genome suggests that it is involved in iron metabolism; it is located downstream of the *feuABC* operon and is Fur regulated (39, 40). YbbA also demonstrates sequence identity with E. coli esterases possessing trilactone hydrolyzation activity (45, 53). However, YbbA esterase activity has not been demonstrated *in vitro*.

To determine whether *ybbA* is required for enterobactin-mediated promotion of sporulation gene expression, we constructed a sporulation reporter strain with a *ybbA* mutation (P_*sspB*_-*yfp*, *ybbA*::*kan*). This strain responded to enterobactin significantly less than the wild-type P_*sspB*_-*yfp* strain (Fig. 7A). To demonstrate that the *ybbA* gene was responsible for this effect, we complemented the *ybbA* mutation by expressing *ybbA* under a xylose-inducible promoter (P_*xyl*_) at a neutral site on the B. subtilis chromosome (*lacA*). This complemented strain exhibited restored enterobactin-mediated promotion of sporulation gene expression (Fig. 7B). (The addition of xylose to these assay plates appeared to decrease the response of the P_*sspB*_-*yfp* reporter to enterobactin overall.) This result suggests that *ybbA* is required for enterobactin to activate sporulation gene expression in B. subtilis. Based on these results and its genomic context, we propose that YbbA may act as an esterase that hydrolyzes enterobactin and allows it to act as a sporulation cue.

To test whether *ybbA* is required by B. subtilis for utilization of enterobactin-bound iron under iron-starved conditions, we performed a modification of an established disc diffusion assay (43). Various B. subtilis mutants were overlaid onto agar plates containing 2,2′-dipyridyl, to determine whether they could grow using the natively produced bacillibactin as their source for iron or could grow only when supplemented with enterobactin. As summarized in Table 2, the *feuA* mutant (which is unable to take up bacillibactin or enterobactin) could not grow in the presence of the iron chelator 2,2′-dipyridyl, regardless of whether enterobactin was available, while the *dhbA* mutant (which is unable to synthesize bacillibactin) could grow only when provided with enterobactin; these results are consistent with previous findings (39, 43). In contrast, while the *ybbA* mutant could grow in the entire overlay, the *dhbA ybbA* double mutant could not grow either with or without enterobactin supplementation (Table 2). These results suggest that a *ybbA* mutant is capable of using bacillibactin as a source for iron but *ybbA* is required for B. subtilis to use enterobactin for iron acquisition when bacillibactin is not available.

**TABLE 2:** Summary of disc diffusion growth assays, demonstrating which strains were able to use enterobactin under iron-limited conditions (*n* ≥ 2)

Strain	Gene function	Growth	
		With enterobactin (near disc)	Without enterobactin (far from disc)
Wild type		+	+
*feuA*	Siderophore uptake	−	−
*dhbA*	Bacillibactin biosynthesis	+	−
*ybbA*	Putative esterase	+	+
*dhbA ybb*A	See above	−	−

### Other siderophores accelerate sporulation in B. subtilis.

**FIG 8:**
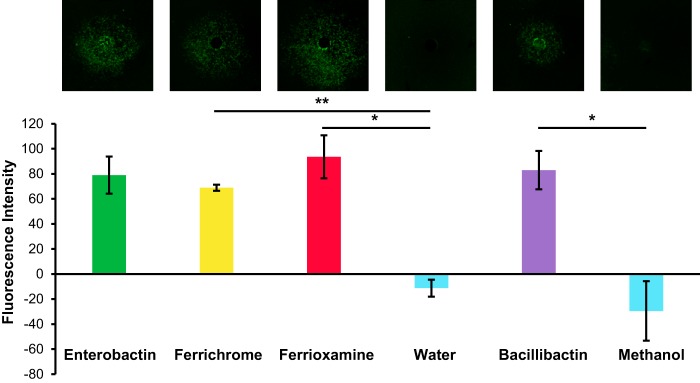
Multiple siderophores promote sporulation gene expression in B. subtilis. The fluorescence intensity of wild-type B. subtilis P_*sspB*_-*yfp* microcolony lawns in response to 10 μM ferrichrome, 10 μM ferrioxamine E, or 10 μM bacillibactin or the corresponding solvent for each compound was quantified; representative fluorescence images of the B. subtilis microcolony lawns (false-colored green) are shown at the top. All three siderophores (ferrichrome, ferrioxamine E, and bacillibactin) significantly promoted sporulation gene expression (*n* ≥ 3). Error bars represent standard deviations. *, *P* < 0.05; **, *P* < 0.000005.

B. subtilis is able to pirate multiple classes of siderophores using its diverse range of siderophore uptake systems (28). To determine whether promotion of sporulation gene expression was specific to enterobactin or was a general response to xenosiderophores, we tested the activities of multiple siderophores in our microcolony lawn assay. We added 10 μM solutions of the xenosiderophores ferrichrome and ferrioxamine E to wells that had been cored in agar plates containing microcolony lawns of the P_*sspB*_-*yfp* reporter, and we imaged the plates for fluorescence. Both ferrichrome and ferrioxamine E treatment resulted in promotion of sporulation gene expression (Fig. 8). We also tested whether the B. subtilis siderophore bacillibactin could promote sporulation in B. subtilis, and we similarly observed activation of sporulation gene expression (Fig. 8). Therefore, both endogenous and foreign siderophores are capable of stimulating B. subtilis cells to sporulate early.

### The *besA* gene is required for bacillibactin-mediated acceleration of sporulation gene expression.

**FIG 9:**
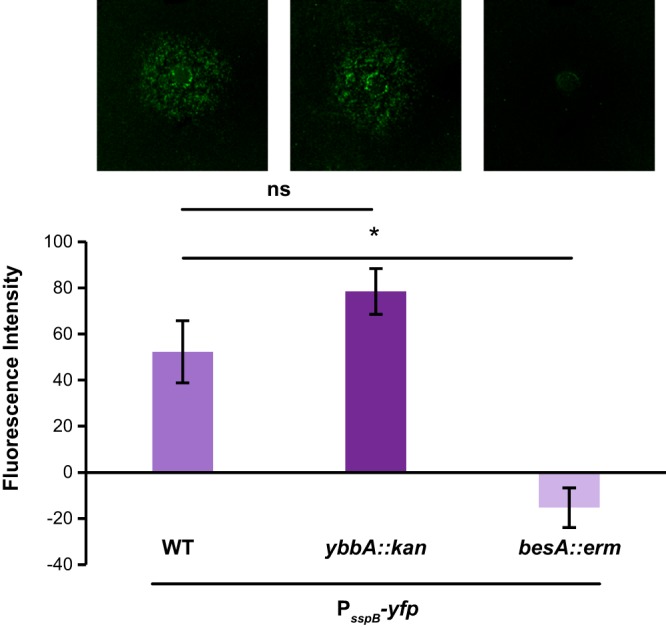
The *ybbA* gene is specific for enterobactin and is not required for bacillibactin to promote sporulation gene expression. The fluorescence intensity of *besA*, *ybbA*, or wild-type (WT) B. subtilis P_*sspB*_-*yfp* microcolony lawns in response to 10 μM bacillibactin was quantified, and the corresponding fluorescence images of the lawns (false-colored green) are shown at the top. There was no significant difference in sporulation gene expression on wild-type or *ybbA* microcolony lawns with bacillibactin. In contrast, sporulation gene expression was significantly decreased on *besA* microcolony lawns (*n* = 3). Error bars represent standard deviations. *, *P* < 0.05; ns, not significant.

We demonstrated that the putative esterase gene *ybbA* is required for enterobactin to promote sporulation gene expression. We next wanted to establish whether *ybbA* is required for the sporulation stimulation activity of bacillibactin. To determine whether bacillibactin-mediated promotion of sporulation gene expression requires either *besA* or *ybbA* activity, we monitored sporulation using reporter strains that lacked one of those two genes (i.e., P_*sspB*_-*yfp*, *ybbA*::*kan* or P_*sspB*_-*yfp*, *besA*::*erm*). Bacillibactin was added to wells that had been cored in agar plates, and microcolony lawns of the two strains were plated. After imaging the plates for fluorescence, we found that sporulation gene expression was significantly decreased on the *besA* mutant lawns compared to the wild-type reporter lawns treated with an equivalent concentration of bacillibactin (Fig. 9). There was no significant difference in P_*sspB*_-*yfp* fluorescence between the *ybbA* mutant lawns treated with bacillibactin and the wild-type reporter lawns (Fig. 9). From these results, we conclude that *ybbA* is specific for enterobactin-mediated sporulation acceleration, while *besA* is required for bacillibactin-mediated sporulation acceleration.

## DISCUSSION

In natural environments, bacteria are constantly interacting with each other either directly or via secreted molecules (7). In this work, we have identified an interaction between B. subtilis and E. coli that affects sporulation in B. subtilis. While B. subtilis is commonly found in the soil, it has also been isolated from, and can grow within, the mammalian gut (54–56). Similarly, E. coli is found in the gut. Therefore, an interaction between B. subtilis and E. coli may be biologically relevant, as the environmental niches of these two bacteria overlap. In addition, we have shown that ferrichrome (produced by multiple fungi [28, 57]) and ferrioxamine E (produced by streptomycetes [28, 58]) also promote sporulation in B. subtilis. Thus, multiple microbes from environments where B. subtilis exists in nature secrete siderophores that alter the physiological development of B. subtilis.

Siderophores have long been thought of exclusively as iron chelators (24). However, there is increasing evidence that siderophores can play roles other than scavenging iron, including transporting other metals, protecting cells against oxidative stress, and acting as cellular signals that modulate bacterial development (59–61). For example, Pseudomonas aeruginosa produces siderophores that affect its own biofilm formation (62). In addition, in a coculture between Streptomyces coelicolor and an Amycolatopsis strain, the presence of an *Amycolatopsis*-produced siderophore led to a competition for iron that inhibited the development of aerial hyphae and spores in S. coelicolor (26). Our data add to these examples by demonstrating that siderophores can also act as interspecies cues that accelerate the development of spores in B. subtilis.

Our data suggest that it is the increased level of iron within the cells that leads to sporulation in B. subtilis; both the uptake (by *feuA*) and the putative hydrolysis (by *ybbA*) of enterobactin appear to be required for promotion of sporulation. Because sporulation typically occurs in response to starvation in B. subtilis, it is interesting that excess availability of this mineral nutrient also accelerates sporulation. However, this is not unprecedented; multiple reports have established that the divalent metals iron and manganese are required for sporulation under some conditions (63, 64). Although iron is known to be required for sporulation in B. subtilis (65, 66), the mechanistic connection between iron accumulation in the cells and sporulation gene expression remains unclear. A recent publication ascertained that high iron concentrations affected cellular respiration and thus biofilm formation in B. subtilis via the sensor histidine kinase KinB, and the authors postulated that iron might similarly affect sporulation via this mechanism (15). However, we found that enterobactin promotes sporulation in B. subtilis independently of *kinB* (see Fig. S2 in the supplemental material). There may be a connection between the requirement for iron for sporulation and the requirement for manganese. Both manganese and iron bind to PerR, a regulator of peroxide stress responses in B. subtilis, and act as cofactors (41). In addition to controlling genes involved in peroxide stress, PerR positively regulates the *srfA* operon, encoding surfactin biosynthesis (67). Surfactin is required for efficient sporulation in B. subtilis (68). Thus, one hypothesis is that iron obtained by B. subtilis via siderophore piracy may bind PerR and drive surfactin production, leading to an acceleration of spore formation in some cells.

The ability of siderophores to alter cellular differentiation in B. subtilis suggests that complex microbial interspecies interactions are likely in environments such as the soil and the gut. Because B. subtilis possesses a diverse range of siderophore acquisition systems, it has a striking ability to pirate other species' siderophores (28, 31, 32). When “piratable” siderophores are present in low-iron environments, we predict that B. subtilis cells would respond by taking up those siderophores and sporulating. This would be beneficial to B. subtilis because it would allow those spores to survive in the low-iron environments and then germinate when conditions are favorable again. Although sporulation would cause B. subtilis to relinquish its current resources to competitors, this developmental decision could guarantee its long-term survival by allowing B. subtilis to outlast its competitors. Previous work demonstrated that B. subtilis takes a “bet-hedging approach” to sporulation, with only a subpopulation of cells differentiating into spores (69). Our data are consistent with those findings, i.e., enterobactin accelerates sporulation and increases the viable proportion of B. subtilis cells that sporulate, but the entire population does not respond to this cue. Therefore, siderophores appear to modulate this bet-hedging strategy by biasing a larger proportion of the B. subtilis population to differentiate into spores when confronted with high levels of exogenous siderophores in the environment.

In addition to siderophores acting as “telesensors” (secreted cues that allow B. subtilis to respond to environmental conditions) (60), our data suggest the possibility that B. subtilis may use siderophores as a type of quorum-sensing-like cue to alert them to the presence of bacterial competitors. Although B. subtilis takes up enterobactin using the same SBP (FeuA) that it uses for bacillibactin (28, 35, 43, 44) (presumably due to the structural similarities between these two siderophores [43, 44, 70]), B. subtilis requires two distinct genes (*besA* and *ybbA*) to sporulate in response to these structurally related compounds, which may allow B. subtilis to differentiate between self-produced bacillibactin and non-self-produced enterobactin. This could potentially allow B. subtilis to distinguish between environments with low iron availability and those with many bacterial competitors and to modulate its extent of sporulation accordingly.

In contrast, in a low-iron environment containing siderophores that B. subtilis cannot take up, we predict that sporulation could be affected negatively. In our 2,2′-dipyridyl assays, we did not see a significant decrease in sporulation gene expression; however, previous work showed that this iron chelator can decrease sporulation (65). This situation would be more analogous to the previously described interaction between actinomycete strains, in which S. coelicolor sporulation was inhibited when the S. coelicolor was unable to take up a siderophore produced by a competing Amycolatopsis strain (26).

A diverse network of siderophore production and siderophore uptake capabilities likely exists within bacteria coexisting in natural microbial communities. Our data suggest that, in addition to affecting simple survival, these specialized metabolites may also lead to complex interspecies interactions that alter sporulation and thus colony differentiation. The availability and diversity of siderophores present in any particular environmental niche thus may affect not only survival (by determining bacterial access to iron) but also the timing and extent of sporulation of some members of the community.

## MATERIALS AND METHODS

### Strains and growth conditions.

All strains used in this study are listed in Table 1. For routine growth, strains were grown in liquid LB-Lennox medium (10 g/liter tryptone, 5 g/liter yeast extract, 5 g/liter NaCl). For plate-based assays, strains were grown on 0.1× LB-Lennox medium and 100 mM MOPS (pH 7) with 1.5% agar. For *ybbA* complementation, 0.1% xylose was added to the plates. Plates were routinely placed at 30°C for growth; for sporulation assays, plates were grown at 24°C. The following antibiotics were used (final concentrations): erythromycin, 1 μg/ml, plus lincomycin, 25 μg/ml (for the *mls* gene); spectinomycin, 100 μg/ml; kanamycin, 15 μg/ml.

### Construction of *ybbA* complementation strain.

To complement the *ybbA* mutant, the entire *ybbA* gene was amplified from B. subtilis NCBI3610 DNA with the primers GGAAGAGTGCGGCCGCCCGCTCATAGGGTTGAGCGGTTTTTTC and CTAAAAATCAAAGGGGGAAATGGTTGAAAGGATCTCTTTCAGAACAC, for Gibson assembly with pAX01 (obtained from the Bacillus Genetic Stock Center). pAX01 was digested with SacII and BamHI. The *ybbA* PCR product was inserted between the SacII and BamHI sites in pAX01 to generate pAX01-*ybbA*, in which the xylose-inducible promoter (P_*xyl*_) drives *ybbA* expression, using an E5510 Gibson assembly cloning kit (NEB). pAX01-*ybbA* was transformed into 5α E. coli (NEB), and transformants were selected for ampicillin resistance. The ampicillin-resistant transformants were checked by colony PCR, and the plasmids from the positive hits were sequenced using the Gibson primers, an internal *ybbA* primer, and a downstream *lacA* primer. Constructs were introduced into the *lacA* locus of B. subtilis 168 by natural competence, after digestion with PflFI. Then, the insert was transferred to the recipient B. subtilis NCBI3610 *ybbA*::*kan*, *amyE*::P_*sspB*_-*yfp* strain by SPP1 phage-mediated transduction (71).

### Siderophores.

Enterobactin, ferrioxamine E, and ferrichrome were purchased from Sigma-Aldrich, and bacillibactin was purchased from EMC Microcollections GmbH (Tuebingen, Germany).

### SPP1 phage transduction.

B. subtilis 168 mutants were purchased from the Bacillus Genetic Stock Center (except for the *ybbA* mutant, which was generously donated by John Helmann, Cornell University) and were used to make the corresponding mutants in B. subtilis 3610 by using SPP1 phage transduction (71). The donor strains (B. subtilis 168 mutants) were grown at 37°C in tryptone-yeast (TY) broth (10 g/liter tryptone, 5 g/liter yeast extract, 5 g/liter NaCl, 10 mM MgSO_4_, 100 mM MnSO_4_) to the late lag/early stationary phase. Cells were infected with SPP1 phage stock at various dilutions, plated on TY plates in an overlay of 0.5% TY soft top agar, and incubated at 37°C for 8 to 16 h. Phage plaques from the donor strain infections were then collected from the TY plates by harvesting and centrifuging the top agar. Phage lysates were filter sterilized using a 0.45-μm filter and stored in 10 mM MgSO_4_ containing 25 μg/ml DNase I at 4°C, with a few drops of chloroform added to prevent growth. The SPP1 phage lysates from the B. subtilis 168 mutants were used to infect the recipient strain. The recipient strain was grown at 37°C in TY broth to the late lag/early stationary phase and then infected with the phage lysates from the B. subtilis 168 mutants and incubated at 37°C for 30 min. Cells were pelleted in a clinical centrifuge, resuspended in 300 μl of the supernatant, and plated on LB-Lennox agar medium with 10 mM citrate and the corresponding antibiotic for the donor strain. Plates were incubated at 37°C for 12 to 24 h. Three colonies per phage transduction were picked and struck out on LB-Lennox agar plates with the corresponding antibiotic for the donor strain. Strains that had the correct antibiotic resistance were struck to singles two more times on LB-Lennox agar plates with the corresponding antibiotic for the donor strain. Strains were then spotted onto 30-ml MSgg agar plates (1.5% Bacto agar, 5 mM potassium phosphate [pH 7], 100 mM MOPS [pH 7], 2 mM MgCl_2_, 700 μM CaCl_2_, 50 μM MnCl_2_, 50 μM FeCl_3_, 1 μM ZnCl_2_, 2 μM thiamine, 0.5% glycerol, 0.5% glutamate) (72) to confirm correct colony morphology.

### Preparation of reporter strain aliquots.

Reporter strain aliquots were prepared using an adaption of an established protocol (46). In brief, reporter strains were grown overnight at 30°C for approximately 12 h. Five milliliters of liquid LB-Lennox cultures were inoculated and rotated at 37°C until cultures reached an OD_600_ of ∼0.5 to 0.6. Cultures were then diluted into 5 ml of fresh liquid LB-Lennox medium to an OD_600_ of 0.02, rotated at 37°C until they reached an OD_600_ of ∼0.4, and then diluted with 10 ml of liquid LB-Lennox medium and 5 ml of 80% glycerol to obtain a solution with a final concentration of 20% glycerol (vol/vol). Portions (250 μl) of the cultures were aliquoted into 0.5-μl microcentrifuge tubes and frozen at −80°C. CFU per milliliter were calculated for the aliquots, for use in the sporulation assays.

### Sporulation microcolony agar assay.

To monitor sporulation promotion, a previously established coculture assay using a fluorescent transcriptional reporter was used (46). Briefly, 50 μl of the relevant B. subtilis P_*sspB*_-yfp reporter strains, at a concentration of ∼5 × 10^−4^ CFU/ml, was plated on 0.1× LB medium-100 mM MOPS (pH 7) agar plates to form microcolony lawns, and allowed to dry.

To test the sporulation promotion activity of bacterial samples, resuspended cells were normalized to an OD_600_ of 0.5 and 1 μl of the cell suspensions was spotted onto the microcolony lawns. The plates were incubated at 24°C, and fluorescence was examined from ∼24 to 32 h using a Typhoon Trio+ variable mode imager (excitation, 532 nm; emission, 526 nm; photomultiplier tube setting, 500; resolution, 200 μm; scan height, 3 mm).

To test the sporulation promotion activity of liquid solutions, microcolony lawns of the reporter strain were plated, and wells were cored in the agar using autoclaved drinking straws approximately 6 mm in diameter. Wells were filled with 80 μl of conditioned medium or liquid solution of either enterobactin, 2,2′-dipyridyl, ferrichrome, ferrioxamine E, bacillibactin, or FeSO_4_. (The solution absorbs into the agar around the well, allowing much larger volumes to be tested than could be spotted on top of the plate.) When necessary, wells were subsequently filled with 120 μl of molten agar; after the agar solidified, microcolony lawns of the reporter strain could be plated on top. Plates were incubated at 24°C, and fluorescence was examined from ∼24 to 30 h using the Typhoon Trio+ variable mode imager. Enterobactin and 2,2′-dipyridyl were tested at 10 μM and 100 μM, and FeSO_4_ was tested at 10 μM, 100 μM, and 1 mM. After these multiple concentrations were tested, all siderophores (enterobactin, bacillibactin, ferrichrome, and ferrioxamine E) were used at 10 μM and FeSO_4_ was used at 1 mM.

### Conditioned medium.

LB-Lennox liquid cultures (5 ml) were inoculated with cells, and one tube of broth was left uninoculated for unconditioned medium. Cultures were grown for 24 h at 37°C, with rotation. Cells were pelleted by centrifugation of the cultures at 4,000 × *g* for 20 min at 4°C, filter sterilized with a 0.22-μm Steriflip filter (Millipore), and stored at −20°C until use.

### Fluorescence quantification.

Typhoon data files (.gel) were loaded into Metamorph (version 7.1), and fluorescence was quantified as described previously by Bleich et al. (5), except that here values were not normalized to the maximum intensity or scaled to the average background intensity. Briefly, for each plate, four concentric rings were outlined around the colony spot or well. The regions were defined as follows: (i) the innermost region, i.e., the cored well or colony spot itself, (ii) the microcolonies directly surrounding the well (potential area of induction), (iii) a spacer region, and (iv) outermost distant microcolonies (background). The difference between the integrated intensities of the first and second regions was divided by the difference between the areas of the first and second regions, which gave us the integrated intensity per area for the region of fluorescence induction (induction fluorescence intensity). The background fluorescence intensity was similarly calculated using the data from the third and fourth regions. The background fluorescence intensity was subtracted from the induction fluorescence intensity to take into account any background fluorescence inherent to the assay and to account for variability between plates.

### Keio Collection screen for sporulation activation.

Plates (96 wells) were filled with 150 μl of 1× LB medium per well. Wells were inoculated with frozen glycerol stocks of E. coli mutants from the Keio Collection by using a 96-well plate pin tool. Cultures were incubated in a 37°C shaker for approximately 18 h. Following incubation, the OD_600_ values of the cultures were determined in a plate reader. Culture aliquots (5 μl) were removed from the wells and spotted onto microcolony lawns of B. subtilis P_*sspB*_-yfp; 4 samples were spotted per plate. Plates were incubated at 24°C, and fluorescence was examined from ∼24 to 32 h.

### Microscopic imaging.

The sporulation microcolony agar assays were set up with the B. subtilis P_*sspB*_-yfp fluorescence reporter strain as described above. At 24 h, samples were taken for imaging from two plates treated with enterobactin and two plates treated with solvent. A hand-driven cork borer (diameter, 2.3 cm) was used to remove a disc of agar from around the well in each plate. This approach captured the cells from the microcolony lawn that were closest to the well and thus had been exposed to the highest concentrations of the added solutions. Each agar disc was placed in a new sterile petri dish, 250 μl of LB medium was added, and a bent sterile pipette tip was used to scrape the cells off the agar disc and resuspend them in the LB medium. The resulting cell suspensions were placed in 1.5-ml tubes. The agar discs were washed once more with 250 μl of LB medium, and the wash was pooled with the initial cell suspension from each plate. To make sure that there were no large clumps of cells, samples were sheared using a 23-gauge needle and a 1-ml syringe; 7-μl samples were then mounted onto thin agar slabs on glass slides, and a glass coverslip was placed over them. Samples were imaged with a Nikon Eclipse 80i microscope using a 40× nonoil objective. Both bright-field and fluorescence images were obtained for each field of view and used for analysis. For representative images, the 40× oil objective was used to obtain differential interference contrast (DIC) and fluorescence images.

### Microscopic image analysis.

Three biological replicates were performed for each treatment (enterobactin or solvent control), with two technical replicates each, and five representative images were selected from each technical replicate. More than 4,800 cells were counted for each treatment. To quantify the total number of cells per image, cells were counted by hand in ImageJ using the pointer tool. To quantify the number of fluorescent cells per image, the fluorescence images were loaded into ImageJ and converted into 8-bit images. The threshold levels were adjusted to minimize the background; for particle analysis, the lower threshold limit was set to 30 and the upper limit was set to 255. The particle analysis tool was set to count particles in the size range of 5 to infinity. We then calculated the percentage of total cells that were fluorescent per image and averaged those values across the biological replicates.

### Spore counts.

The sporulation microcolony agar assays were set up with the B. subtilis P_*sspB*_-yfp fluorescence reporter strain as described above. Wells were cored into agar plates and either 80 μl of 10 μM enterobactin or 80 μl of 9:1 acetonitrile/distilled water (solvent control) was added to the well. At multiple time points (30, 36, and 54 h), samples were taken for spore counts from two plates treated with enterobactin and two plates treated with solvent. A hand-driven cork borer (diameter, 2.3 cm) was used to remove a disc of agar around the well in each plate. Each agar disc was placed in a new sterile petri dish, and 500 μl of LB medium was added to resuspend the cells. A bent sterile pipette tip was used to scrape the cells off each disc. The resulting LB cell suspensions were placed in 1.5-ml tubes. The agar discs were washed two times with 250 μl of LB medium, and the washes were pooled with the initial cell suspension from each plate. Samples were sonicated using a QSonica sonicator, for a total processing time of 12 s; each pulse was 1 s long, with a 1-s pause between pulses, at an amplitude of 20%. We confirmed that the sonication treatment did not lyse cells by measuring the levels of the cytoplasmic protein σ^A^ in the cell pellet and supernatant via Western blotting, as described previously (73). Aliquots (500 μl) of the cell suspension samples were heat treated, and 500-μl portions were left untreated. The heat-treated samples were heated at 80°C for 20 min. Both heat-treated and untreated samples were serially diluted with LB medium. The 10^0^ to 10^−6^ dilutions were plated on 1× LB-Lennox agar plates using a drip dilution technique, with a 5-μl volume for each dilution. Plates were incubated overnight at 30°C for approximately 16 h. Colonies from each dilution were counted, and the CFU per milliliter were calculated for each sample. The heat-treated plates were used to calculate the total number of spores, while the untreated plates were used to calculate the total number of cells; together, these data allowed us to calculate the percentage of spores among the total cells in each sample.

### Disc diffusion growth stimulation assay.

The protocol for the growth stimulation assay was adapted from the method described by Abergel et al. (43). Briefly, strains were grown in 5 ml of LB-Lennox liquid medium until the late exponential/early stationary phase. The strains tested in the assay were B. subtilis 3610, ES809, ES810, ES812, and ES814. Cells were centrifuged at 4,000 × *g* for 5 min, and the supernatant was removed. Cells were washed twice with Tris-EDTA buffer (pH 8) and resuspended in 150 μl distilled water. Cells were diluted 1:100 into 3 ml of 0.5% molten agarose and vortex-mixed briefly. The cell suspensions were overlaid onto 1× LB-Lennox medium-0.5 mM 2,2′-dipyridyl agar plates. Sterile discs (6 mm) infused with either 20 μl of 10 μM enterobactin or 20 μl of a 9:1 acetonitrile/water mixture were put on the overlays. Plates were incubated overnight at 37°C for 16 h and then were scored for growth.

### Statistics.

All *P* values were determined using Student's *t* test, with *n* values of at least 3.

### Images.

Images of the microcolony lawns were obtained using a Zeiss SteREO Discovery.V8 dissecting stereomicroscope with a 1× objective. The brightness and contrast of the images were linearly adjusted using Adobe Photoshop CS6.

## Supplementary Material

Supplemental material
