# Supplementary material for: Pirated Siderophores Promote Sporulation in Bacillus subtilis
Source: Appl Environ Microbiol. 2017 May 1;83(10):e03293-16. doi: 10.1128/AEM.03293-16 (PMC5411514; doi:10.1128/AEM.03293-16)
Supplement: Supplemental material [file supp_83_10_e03293-16__index.html]

Supplemental material 

# Pirated Siderophores Promote Sporulation in Bacillus subtilis

## Supplemental material

- Supplemental file 1 -

  Enterobactin does not affect growth of *B. subtilis* (Fig. S1); sporulation gene promotion by enterobactin is independent of KinB (Fig. S2); summary of results from the Keio collection screen (Table S1).

  PDF, 735K
