# Supplementary material for: Pirated Siderophores Promote Sporulation in Bacillus subtilis
Source: Appl Environ Microbiol. 2017 May 1;83(10):e03293-16. doi: 10.1128/AEM.03293-16 (PMC5411514; doi:10.1128/AEM.03293-16)
Supplement: Supplemental material [file AEM.03293-16_zam999117827s1.pdf]

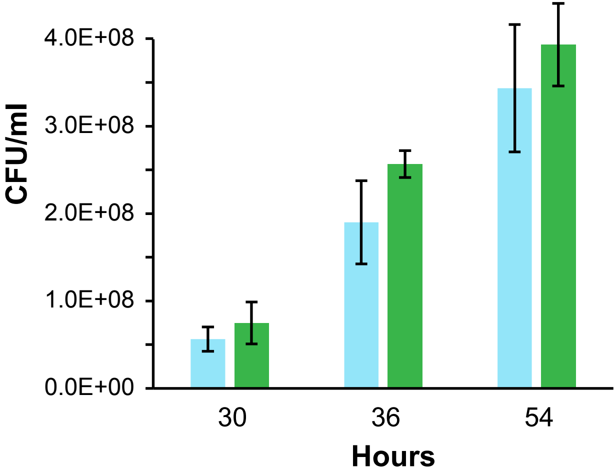

FIG S1
Enterobactin does not affect growth of *B. subtilis*. The total CFU/ml calculated for *B. subtilis* cells treated with 10 μM enterobactin (green) or solvent (blue) at 30, 36, and 54 hours. Samples were taken from microcolony agar assay plates grown in the presence or absence of enterobactin and non-heat treated cells were plated to determine total number of cells. No significant differences were observed between samples treated with enterobactin compared to untreated samples (n=3). Error bars represent standard deviations.

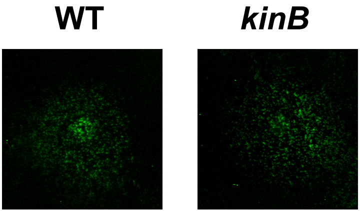

FIG S2

Sporulation gene promotion by enterobactin is independent of KinB. Representative fluorescence images of wild-type and *kinB B. subtilis* P_{sspB}-*yfp* micro-colony lawns on agar plates with wells containing 10 μM enterobactin. Sporulation gene promotion is observed in both wild-type and the *kinB* mutant.

Table S1

Summary of results from the Keio Collection screen. Each mutant within the collection was spotted onto B. subtilis PsspB-yfp microcolony lawns and imaged for fluorescence. Sporulation gene stimulation for each mutant was then noted as either no, low, high, or normal promotion. The table lists all mutants that altered sporulation gene expression compared to wildtype and the corresponding GO terms for each gene where applicable (GO term designations obtained from EcoCyc (Gehring AM, Bradley KA, Walsh CT, *Biochemistry,* 36:8495–503, 1997).

Plate/Well	No induction	Low induction	High induction	Gene	GO terms (biological processes)
P1 A2		x		*bglG*	GO:0045893 - positive regulation of transcription, DNA-templated<br>GO:0006355 - regulation of transcription, DNA-templated<br>GO:0006351 - transcription, DNA-templated
P1 A9		x		*tyrR*	GO:0008152 - metabolic process<br>GO:0006351 - transcription, DNA-templated<br>GO:0045892 - negative regulation of transcription, DNA-templated<br>GO:0019439 - aromatic compound catabolic process<br>GO:0006355 - regulation of transcription, DNA-templated<br>GO:0000160 - phosphorelay signal transduction system
P1 A10		x		*rcsC*	GO:0023014 - signal transduction by protein phosphorylation<br>GO:0018106 - peptidyl-histidine phosphorylation<br>GO:0016310 - phosphorylation<br>GO:0007165 - signal transduction<br>GO:0006355 - regulation of transcription, DNA-templated<br>GO:0046777 - protein autophosphorylation<br>GO:0044010 - single-species biofilm formation<br>GO:0071470 - cellular response to osmotic stress<br>GO:0000160 - phosphorelay signal transduction system
P1 A11		x		*aceF*	GO:0055114 - oxidation-reduction process<br>GO:0008152 - metabolic process<br>GO:0006090 - pyruvate metabolic process<br>GO:0006086 - acetyl-CoA biosynthetic process from pyruvate<br>GO:0006096 - glycolytic process
P1 B2		x		*btuR*	GO:0006779 - porphyrin-containing compound biosynthetic process<br>GO:0019250 - aerobic cobalamin biosynthetic process<br>GO:0009236 - cobalamin biosynthetic process
P1 B9			x	*uidR*	GO:0045892 - negative regulation of transcription, DNA-templated<br>GO:0006355 - regulation of transcription, DNA-templated<br>GO:0006351 - transcription, DNA-templated
P1 C6		x		*melR*	GO:0006351 - transcription, DNA-templated<br>GO:0006355 - regulation of transcription, DNA-templated
P1 C7		x		*nhaR*	GO:0006351 - transcription, DNA-templated<br>GO:0006355 - regulation of transcription, DNA-templated
P1 C8		x		*soxR*	GO:0006355 - regulation of transcription, DNA-templated<br>GO:0006979 - response to oxidative stress<br>GO:0006351 - transcription, DNA-templated
P1 C9		x		*uxuR*	GO:0006355 - regulation of transcription, DNA-templated<br>GO:0006351 - transcription, DNA-templated
P1 D4	x			*galS*	GO:0006351 - transcription, DNA-templated<br>GO:0006355 - regulation of transcription, DNA-templated
P1 E1	x			*appY*	GO:0006355 - regulation of transcription, DNA-templated<br>GO:0006351 - transcription, DNA-templated<br>GO:0016036 - cellular response to phosphate starvation<br>GO:0045893 - positive regulation of transcription, DNA-templated
P1 E2	x			*chbR*	GO:0006355 - regulation of transcription, DNA-templated<br>GO:0006351 - transcription, DNA-templated<br>GO:0045892 - negative regulation of transcription, DNA-templated
P1 E5		x		*leuO*	GO:0006351 - transcription, DNA-templated<br>GO:0006355 - regulation of transcription, DNA-templated
P1 E6	x			*metR*	GO:0006351 - transcription, DNA-templated<br>GO:0008652 - cellular amino acid biosynthetic process<br>GO:0006355 - regulation of transcription, DNA-templated<br>GO:0009086 - methionine biosynthetic process
P1 E9		x		*zntR*	GO:0006355 - regulation of transcription, DNA-templated<br>GO:0006351 - transcription, DNA-templated<br>GO:0045893 - positive regulation of transcription, DNA-templated
P1 E10		x		*rbsR*	GO:0045893 - positive regulation of transcription, DNA-templated<br>GO:0006355 - regulation of transcription, DNA-templated<br>GO:0006351 - transcription, DNA-templated

P1 F1		x		*argR*	GO:0006355 - regulation of transcription, DNA-templated<br>GO:0006351 - transcription, DNA-templated<br>GO:0006310 - DNA recombination<br>GO:0051259 - protein oligomerization<br>GO:0006525 - arginine metabolic process<br>GO:2000143 - negative regulation of DNA-templated transcription, initiation<br>GO:0042150 - plasmid recombination<br>GO:0008652 - cellular amino acid biosynthetic process<br>GO:0006526 - arginine biosynthetic process
P1 F3		x		*envY*	GO:0009266 - response to temperature stimulus<br>GO:0006355 - regulation of transcription, DNA-templated<br>GO:0006351 - transcription, DNA-templated
P1 F6		x		*mhpR*	GO:0045892 - negative regulation of transcription, DNA-templated<br>GO:0006355 - regulation of transcription, DNA-templated<br>GO:0019439 - aromatic compound catabolic process<br>GO:0006351 - transcription, DNA-templated
P1 F10		x		*sdiA*	GO:0051301 - cell division<br>GO:0006355 - regulation of transcription, DNA-templated<br>GO:0006351 - transcription, DNA-templated<br>GO:0032467 - positive regulation of cytokinesis<br>GO:2000144 - positive regulation of DNA-templated transcription, initiation<br>GO:0007049 - cell cycle
P1 F11	x			*cyoB*	GO:0055114 - oxidation-reduction process<br>GO:0015992 - proton transport<br>GO:0009060 - aerobic respiration<br>GO:0006811 - ion transport<br>GO:0015990 - electron transport coupled proton transport<br>GO:0019646 - aerobic electron transport chain
P1 F12	x			*sucB*	GO:0008152 - metabolic process<br>GO:0033512 - L-lysine catabolic process to acetyl-CoA via saccharopine<br>GO:0006099 - tricarboxylic acid cycle
P1 G1		x		*asnC*	GO:0006355 - regulation of transcription, DNA-templated<br>GO:0006351 - transcription, DNA-templated<br>GO:0045893 - positive regulation of transcription, DNA-templated<br>GO:0045892 - negative regulation of transcription, DNA-templated<br>GO:0043200 - response to amino acid
P1 G2		x		*csgD*	GO:0006355 - regulation of transcription, DNA-templated<br>GO:0006351 - transcription, DNA-templated<br>GO:1900190 - regulation of single-species biofilm formation<br>GO:2000144 - positive regulation of DNA-templated transcription, initiation
P1 G3		x		*fadR*	GO:0045723 - positive regulation of fatty acid biosynthetic process<br>GO:0006631 - fatty acid metabolic process<br>GO:0006629 - lipid metabolic process<br>GO:0019217 - regulation of fatty acid metabolic process<br>GO:0019395 - fatty acid oxidation<br>GO:0045892 - negative regulation of transcription, DNA-templated<br>GO:0045893 - positive regulation of transcription, DNA-templated<br>GO:0006355 - regulation of transcription, DNA-templated<br>GO:0006351 - transcription, DNA-templated
P1 G10			x	*srlR*	GO:0006355 - regulation of transcription, DNA-templated<br>GO:0006351 - transcription, DNA-templated<br>GO:0045892 - negative regulation of transcription, DNA-templated<br>GO:0009401 - phosphoenolpyruvate-dependent sugar phosphotransferase system
P1 H1	x			*betI*	GO:0006355 - regulation of transcription, DNA-templated<br>GO:0006351 - transcription, DNA-templated<br>GO:0019285 - glycine betaine biosynthetic process from choline<br>GO:0045892 - negative regulation of transcription, DNA-templated<br>GO:0006970 - response to osmotic stress
P1 H2		x		*cynR*	GO:0006355 - regulation of transcription, DNA-templated<br>GO:0006351 - transcription, DNA-templated
P1 H3		x		*fis*	GO:0006351 - transcription, DNA-templated<br>GO:0034401 - chromatin organization involved in regulation of transcription<br>GO:0006355 - regulation of transcription, DNA-templated
P1 H4	x			*hupA*	GO:0030261 - chromosome condensation<br>GO:0006974 - cellular response to DNA damage stimulus<br>GO:0006351 - transcription, DNA-templated
P1 H5		x		*malI*	GO:0006355 - regulation of transcription, DNA-templated<br>GO:0006351 - transcription, DNA-templated<br>GO:0045892 - negative regulation of transcription, DNA-templated

P1 H8		x		*trpR*	GO:0006351 - transcription, DNA-templated<br>GO:0045892 - negative regulation of transcription, DNA-templated<br>GO:0006355 - regulation of transcription, DNA-templated
P3 A2			x	*adhE*	GO:0008152 - metabolic process<br>GO:0006066 - alcohol metabolic process<br>GO:0015976 - carbon utilization<br>GO:0006115 - ethanol biosynthetic process<br>GO:0055114 - oxidation-reduction process
P3 A6		x		*mdh*	GO:0019752 - carboxylic acid metabolic process<br>GO:0055114 - oxidation-reduction process<br>GO:0005975 - carbohydrate metabolic process<br>GO:0006108 - malate metabolic process<br>GO:0006096 - glycolytic process<br>GO:0006099 - tricarboxylic acid cycle<br>GO:0009061 - anaerobic respiration<br>GO:0006113 - fermentation
P3 B4		x		*nuoK*	GO:0055114 - oxidation-reduction process<br>GO:0042773 - ATP synthesis coupled electron transport<br>GO:0006810 - transport
P3 B5		x		*nuoA*	GO:0055114 - oxidation-reduction process<br>GO:0006810 - transport
P3 B6			x	*yhfW*	GO:0043094 - cellular metabolic compound salvage<br>GO:0009117 - nucleotide metabolic process<br>GO:0008152 - metabolic process
P3 B8	x			*frdC*	GO:0044780 - bacterial-type flagellum assembly<br>GO:0001539 - cilium or flagellum-dependent cell motility<br>GO:0006810 - transport<br>GO:0009061 - anaerobic respiration<br>GO:0006113 - fermentation
P3 B12		x		*atoC*	GO:0010967 - regulation of polyamine biosynthetic process<br>GO:0043086 - negative regulation of catalytic activity<br>GO:0006355 - regulation of transcription, DNA-templated<br>GO:0006351 - transcription, DNA-templated<br>GO:0045893 - positive regulation of transcription, DNA-templated<br>GO:0000160 - phosphorelay signal transduction system
P3 C4		x		*nuoJ*	GO:0055114 - oxidation-reduction process
P3 C9			x	*citB*	GO:0009605 - response to external stimulus<br>GO:0006355 - regulation of transcription, DNA-templated<br>GO:0000160 - phosphorelay signal transduction system<br>GO:0006351 - transcription, DNA-templated
P3 C11			x	*uvrY*	GO:0006355 - regulation of transcription, DNA-templated<br>GO:0006351 - transcription, DNA-templated<br>GO:0000160 - phosphorelay signal transduction system
P3 D7		x		*aceB*	GO:0006099 - tricarboxylic acid cycle<br>GO:0006097 - glyoxylate cycle
P3 E5	x			*talA*	GO:0005975 - carbohydrate metabolic process<br>GO:0006098 - pentose-phosphate shunt
P3 E6		x		*gpmI*	GO:0006979 - response to oxidative stress<br>GO:0008152 - metabolic process<br>GO:0006007 - glucose catabolic process<br>GO:0006096 - glycolytic process
P3 E7		x		*aceA*	GO:0019752 - carboxylic acid metabolic process<br>GO:0008152 - metabolic process<br>GO:0006099 - tricarboxylic acid cycle<br>GO:0006097 - glyoxylate cycle
P3 E8		x		*deoB*	GO:0009166 - nucleotide catabolic process<br>GO:0009264 - deoxyribonucleotide catabolic process<br>GO:0043094 - cellular metabolic compound salvage<br>GO:0009117 - nucleotide metabolic process<br>GO:0008152 - metabolic process<br>GO:0006974 - cellular response to DNA damage stimulus<br>GO:0006015 - 5-phosphoribose 1-diphosphate biosynthetic process
P3 E12		x		*narQ*	GO:0023014 - signal transduction by protein phosphorylation<br>GO:0018106 - peptidyl-histidine phosphorylation<br>GO:0007165 - signal transduction<br>GO:0042128 - nitrate assimilation<br>GO:0016310 - phosphorylation<br>GO:0071249 - cellular response to nitrate<br>GO:0071250 - cellular response to nitrite<br>GO:0000160 - phosphorelay signal transduction system

P3 F4		x		*nuoG*	GO:0055114 - oxidation-reduction process<br>GO:0042773 - ATP synthesis coupled electron transport<br>GO:0009060 - aerobic respiration
P3 F9		x		*dps*	GO:0006950 - response to stress<br>GO:0030261 - chromosome condensation<br>GO:0042594 - response to starvation<br>GO:0055114 - oxidation-reduction process<br>GO:0006879 - cellular iron ion homeostasis
P3 F12		x		*yfhA*	
P3 G3		x		*nuoN*	GO:0055114 - oxidation-reduction process<br>GO:0015990 - electron transport coupled proton transport<br>GO:0042773 - ATP synthesis coupled electron transport<br>GO:0006810 - transport<br>GO:0009060 - aerobic respiration
P3 H1	x			*icd*	GO:0006979 - response to oxidative stress<br>GO:0055114 - oxidation-reduction process<br>GO:0022900 - electron transport chain<br>GO:0006097 - glyoxylate cycle<br>GO:0006099 - tricarboxylic acid cycle
P3 H4	x			*nuoE*	GO:0022904 - respiratory electron transport chain<br>GO:0055114 - oxidation-reduction process
P5 A1			x	*barA*	GO:0023014 - signal transduction by protein phosphorylation<br>GO:0018106 - peptidyl-histidine phosphorylation<br>GO:0016310 - phosphorylation<br>GO:0007165 - signal transduction<br>GO:0006468 - protein phosphorylation<br>GO:0006355 - regulation of transcription, DNA-templated<br>GO:0071310 - cellular response to organic substance<br>GO:0046777 - protein autophosphorylation<br>GO:0042542 - response to hydrogen peroxide<br>GO:0006351 - transcription, DNA-templated<br>GO:0000160 - phosphorelay signal transduction system
P5 A2			x	*uhpA*	GO:0006351 - transcription, DNA-templated<br>GO:0006355 - regulation of transcription, DNA-templated<br>GO:0000160 - phosphorelay signal transduction system
P5 A4			x	*treA*	GO:0008152 - metabolic process<br>GO:0005991 - trehalose metabolic process<br>GO:0006974 - cellular response to DNA damage stimulus<br>GO:0005993 - trehalose catabolic process<br>GO:0071474 - cellular hyperosmotic response
P5 A9		x		*coaE*	GO:0016310 - phosphorylation<br>GO:0015937 - coenzyme A biosynthetic process
P5 B1		x		*qseB*	GO:0010038 - response to metal ion<br>GO:0006355 - regulation of transcription, DNA-templated<br>GO:0000160 - phosphorelay signal transduction system<br>GO:0006351 - transcription, DNA-templated
P5 B3		x		*dcuR*	GO:0045893 - positive regulation of transcription, DNA-templated<br>GO:0009605 - response to external stimulus<br>GO:0006355 - regulation of transcription, DNA-templated<br>GO:0000160 - phosphorelay signal transduction system<br>GO:0006351 - transcription, DNA-templated
P5 B4		x		*cpsB*	GO:0009058 - biosynthetic process<br>GO:0005976 - polysaccharide metabolic process<br>GO:0000271 - polysaccharide biosynthetic process<br>GO:0009298 - GDP-mannose biosynthetic process<br>GO:0006972 - hyperosmotic response<br>GO:0009103 - lipopolysaccharide biosynthetic process<br>GO:0009242 - colanic acid biosynthetic process
P5 B8		x		*tap*	GO:0007165 - signal transduction<br>GO:0006935 - chemotaxis
P5 B9		x		*ykfA*	GO:0000027 - ribosomal large subunit assembly
P5 B12	x			*ypjB*	
P5 C4		x		*rbsB*	GO:0015749 - monosaccharide transport<br>GO:0008643 - carbohydrate transport<br>GO:0006935 - chemotaxis<br>GO:0006810 - transport
P5 C6			x	*yfdF*	
P5 D1		x		*rpoN*	GO:0006352 - DNA-templated transcription, initiation<br>GO:0006525 - arginine metabolic process<br>GO:0006355 - regulation of transcription, DNA-templated

P5 D4		x		*oppA*	GO:0055085 - transmembrane transport<br>GO:0061077 - chaperone-mediated protein folding<br>GO:0009408 - response to heat<br>GO:0015833 - peptide transport<br>GO:0006810 - transport<br>GO:0015031 - protein transport<br>GO:0006857 - oligopeptide transport
P5 D5		x		*lldD*	GO:0055114 - oxidation-reduction process<br>GO:0006089 - lactate metabolic process<br>GO:0019516 - lactate oxidation<br>GO:0009061 - anaerobic respiration<br>GO:0042355 - L-fucose catabolic process<br>GO:0009060 - aerobic respiration
P5 D8		x		*helD*	GO:0032508 - DNA duplex unwinding<br>GO:0023014 - signal transduction by protein phosphorylation<br>GO:0000160 - phosphorelay signal transduction system<br>GO:0006310 - DNA recombination
P5 E1			x	*arcB*	GO:0023014 - signal transduction by protein phosphorylation<br>GO:0018106 - peptidyl-histidine phosphorylation<br>GO:0016310 - phosphorylation<br>GO:0007165 - signal transduction<br>GO:0006355 - regulation of transcription, DNA-templated<br>GO:0006351 - transcription, DNA-templated<br>GO:0046777 - protein autophosphorylation<br>GO:0000160 - phosphorelay signal transduction system
P5 E2			x	*cpxR*	GO:0007155 - cell adhesion<br>GO:0010810 - regulation of cell-substrate adhesion<br>GO:0006355 - regulation of transcription, DNA-templated<br>GO:0006351 - transcription, DNA-templated<br>GO:0045892 - negative regulation of transcription, DNA-templated<br>GO:0045893 - positive regulation of transcription, DNA-templated<br>GO:0000160 - phosphorelay signal transduction system
P5 E5	x			*pgm*	GO:0071704 - organic substance metabolic process<br>GO:0019388 - galactose catabolic process<br>GO:0005978 - glycogen biosynthetic process<br>GO:0005975 - carbohydrate metabolic process<br>GO:0006006 - glucose metabolic process
P5 E6		x		*cusA*	GO:1902601 - silver ion transmembrane transport<br>GO:0006825 - copper ion transport<br>GO:0006812 - cation transport<br>GO:0006811 - ion transport<br>GO:0006810 - transport<br>GO:0006878 - cellular copper ion homeostasis<br>GO:0010272 - response to silver ion<br>GO:0010273 - detoxification of copper ion<br>GO:0015673 - silver ion transport<br>GO:0015679 - plasma membrane copper ion transport<br>GO:0046688 - response to copper ion<br>GO:0060003 - copper ion export
P5 E8	x			*hrpA*	GO:0009451 - RNA modification<br>GO:0006397 - mRNA processing
P5 E9		x		*yahL*	
P5 E11		x		*yfiN*	GO:0036460 - cellular response to cell envelope stress<br>GO:0008152 - metabolic process<br>GO:1902201 - negative regulation of bacterial-type flagellum-dependent cell motility<br>GO:0007165 - signal transduction
P5 E12	x			*yqaD*	GO:0005975 - carbohydrate metabolic process
P5 F2		x		*zraS*	GO:0007165 - signal transduction<br>GO:0023014 - signal transduction by protein phosphorylation<br>GO:0018106 - peptidyl-histidine phosphorylation<br>GO:0016310 - phosphorylation<br>GO:0071294 - cellular response to zinc ion<br>GO:0071284 - cellular response to lead ion<br>GO:0046777 - protein autophosphorylation<br>GO:0000160 - phosphorelay signal transduction system
P5 F3		x		*arcA*	GO:0006351 - transcription, DNA-templated<br>GO:0045892 - negative regulation of transcription, DNA-templated<br>GO:0045893 - positive regulation of transcription, DNA-templated<br>GO:0006355 - regulation of transcription, DNA-templated<br>GO:0000160 - phosphorelay signal transduction system

P5 F4	x			*rnt*	GO:0090503 - RNA phosphodiester bond hydrolysis, exonucleolytic<br>GO:0090501 - RNA phosphodiester bond hydrolysis<br>GO:0090305 - nucleic acid phosphodiester bond hydrolysis<br>GO:0008033 - tRNA processing<br>GO:0006396 - RNA processing<br>GO:0042780 - tRNA 3'-end processing
P5 F7		x		*gspA*	GO:0006810 - transport
P5 F8		x		*moaA*	GO:0009408 - response to heat<br>GO:0006777 - Mo-molybdopterin cofactor biosynthetic process
P5 F9		x		*aceE*	GO:0008152 - metabolic process<br>GO:0055114 - oxidation-reduction process<br>GO:0006096 - glycolytic process
P5 G5		x		*cydB*	GO:0019646 - aerobic electron transport chain<br>GO:0006119 - oxidative phosphorylation<br>GO:0055114 - oxidation-reduction process
P5 G6		x		*fhuA*	GO:0044718 - siderophore transmembrane transport<br>GO:0015891 - siderophore transport<br>GO:0055072 - iron ion homeostasis<br>GO:0006811 - ion transport<br>GO:0006810 - transport
P5 G8		x		*speE*	GO:0006596 - polyamine biosynthetic process<br>GO:0008295 - spermidine biosynthetic process
P5 G9	x			*lpd*	GO:0006979 - response to oxidative stress<br>GO:0045454 - cell redox homeostasis<br>GO:0006103 - 2-oxoglutarate metabolic process<br>GO:0006090 - pyruvate metabolic process<br>GO:0055114 - oxidation-reduction process<br>GO:0006096 - glycolytic process<br>GO:0019464 - glycine decarboxylation via glycine cleavage system
P5 G10		x		*ypdI*	GO:0009242 - colanic acid biosynthetic process
P5 G12	x			*hha*	GO:0006355 - regulation of transcription, DNA-templated<br>GO:0006351 - transcription, DNA-templated<br>GO:0010468 - regulation of gene expression
P5 H1		x		*uhpB*	GO:0006470 - protein dephosphorylation<br>GO:0023014 - signal transduction by protein phosphorylation<br>GO:0018106 - peptidyl-histidine phosphorylation<br>GO:0016310 - phosphorylation<br>GO:0046777 - protein autophosphorylation<br>GO:0000160 - phosphorelay signal transduction system
P5 H6		x		*pstB*	GO:0008152 - metabolic process<br>GO:0006810 - transport<br>GO:0035435 - phosphate ion transmembrane transport<br>GO:0006817 - phosphate ion transport
P5 H9		x		*fbp*	GO:0030388 - fructose 1,6-bisphosphate metabolic process<br>GO:0006002 - fructose 6-phosphate metabolic process<br>GO:0006000 - fructose metabolic process<br>GO:0005986 - sucrose biosynthetic process<br>GO:0006001 - fructose catabolic process<br>GO:0051289 - protein homotetramerization<br>GO:0016311 - dephosphorylation<br>GO:0016051 - carbohydrate biosynthetic process<br>GO:0008152 - metabolic process<br>GO:0005975 - carbohydrate metabolic process<br>GO:0006094 - gluconeogenesis
P7 A3		x		*ybhC*	GO:0008152 - metabolic process<br>GO:0042545 - cell wall modification
P7 A12		x		*pepT*	GO:0043171 - peptide catabolic process<br>GO:0008152 - metabolic process<br>GO:0006518 - peptide metabolic process<br>GO:0006508 - proteolysis
P7 G8		x		*yidZ*	GO:0006355 - regulation of transcription, DNA-templated<br>GO:0006351 - transcription, DNA-templated
P7 G11		x		*pepN*	GO:0043171 - peptide catabolic process<br>GO:0006508 - proteolysis
P9 E1			x	*ptrB*	GO:0006508 - proteolysis
P9 E2			x	*pepB*	GO:0043171 - peptide catabolic process<br>GO:0019538 - protein metabolic process<br>GO:0006508 - proteolysis
P9 E3			x	*yggG*	
P9 E4			x	*degQ*	GO:0051603 - proteolysis involved in cellular protein catabolic process<br>GO:0006508 - proteolysis

P9 G6		x		*pepE*	GO:0006508 - proteolysis
P9 G10		x		*yfcR*	GO:0007155 - cell adhesion
P13 G7		x		*yciC*	
P13 G8		x		*mpaA*	GO:0006508 - proteolysis<br>GO:0009253 - peptidoglycan catabolic process<br>GO:0009050 - glycopeptide catabolic process
P15 C9	x			*ybfQ*	
P15 D4			x	*yaiV*	GO:0006351 - transcription, DNA-templated
P15 E9		x		*ybgE*	
P15 F7			x	*ybdD*	
P15 G5	x			*ybaP*	
P15 G6		x		*ybcO*	
P15 G7		x		*ybdM*	
P15 H6		x		*ybcQ*	GO:0044010 - single-species biofilm formation<br>GO:0060567 - negative regulation of DNA-templated transcription, termination<br>GO:0006355 - regulation of transcription, DNA-templated<br>GO:0031564 - transcription antitermination<br>GO:0006351 - transcription, DNA-templated
P15 H10	x			*ybjN*	GO:1902209 - negative regulation of bacterial-type flagellum assembly<br>GO:0010212 - response to ionizing radiation<br>GO:1902208 - regulation of bacterial-type flagellum assembly<br>GO:1900232 - negative regulation of single-species biofilm formation on inanimate substrate<br>GO:0030308 - negative regulation of cell growth<br>GO:0009297 - pilus assembly<br>GO:1902201 - negative regulation of bacterial-type flagellum-dependent cell motility
P17 A2			x	*ycjX*	
P17 B3		x		*yniA*	GO:0016310 - phosphorylation
P17 B4		x		*yejL*	
P17 E5		x		*yfhJ*	GO:0043086 - negative regulation of catalytic activity<br>GO:0016226 - iron-sulfur cluster assembly
P17 E6		x		*yaaX*	
P17 F11		x		*ybeA*	GO:0032259 - methylation<br>GO:0006364 - rRNA processing<br>GO:0031167 - rRNA methylation<br>GO:0070475 - rRNA base methylation
P17 G3		x		*yeeX*	
P17 G4		x		*yfcN*	
P17 H7	x			*yaeQ*	
P17 H9		x		*yeeS*	
P17 H11	x			*ybeT*	
P17 H12	x			*yedK*	GO:0006508 - proteolysis
P19 A9		x		*mraW*	GO:0032259 - methylation<br>GO:0006364 - rRNA processing<br>GO:0070475 - rRNA base methylation
P19 B1	x			*yegU*	GO:0008152 - metabolic process
P19 B2		x		*yaaH*	GO:0015992 - proton transport<br>GO:0006811 - ion transport<br>GO:0006810 - transport<br>GO:0035433 - acetate transmembrane transport<br>GO:0071422 - succinate transmembrane transport
P19 B3		x		*ybgH*	GO:0015992 - proton transport<br>GO:0015833 - peptide transport<br>GO:0006857 - oligopeptide transport<br>GO:0006810 - transport<br>GO:0015031 - protein transport<br>GO:0042938 - dipeptide transport
P19 B4		x		*ydiU*	
P19 D8		x		*ycdO*	GO:0009411 - response to UV<br>GO:0009636 - response to toxic substance<br>GO:0006979 - response to oxidative stress<br>GO:0046677 - response to antibiotic
P19 D11		x		*iscA*	GO:0097428 - protein maturation by iron-sulfur cluster transfer<br>GO:0006790 - sulfur compound metabolic process<br>GO:0016226 - iron-sulfur cluster assembly
P19 E7		x		*ycaP*	
P19 E8	x			*yebC*	GO:0010212 - response to ionizing radiation<br>GO:0006355 - regulation of transcription, DNA-templated<br>GO:0006351 - transcription, DNA-templated

P19 E11		x		*ygbL*	GO:0019323 - pentose catabolic process
P19 E12	x			*ybhK*	GO:0008360 - regulation of cell shape
P19 G2		x		*ybbY*	GO:0055085 - transmembrane transport<br>GO:0006810 - transport
P19 H3		x		*ydgR*	
P19 H5		x		*ybbB*	
P19 H6		x		*ybhH*	GO:0008152 - metabolic process
P19 H8		x		*yfaE*	GO:0055114 - oxidation-reduction process<br>GO:0006124 - ferredoxin metabolic process
P19 H9		x		*ydjI*	GO:0005975 - carbohydrate metabolic process
P19 H10		x		*yciV*	GO:0090503 - RNA phosphodiester bond hydrolysis, exonucleolytic<br>GO:0090305 - nucleic acid phosphodiester bond hydrolysis<br>GO:0016311 - dephosphorylation<br>GO:0006260 - DNA replication<br>GO:0071897 - DNA biosynthetic process
P19 H11		x		*uspE*	GO:0071973 - bacterial-type flagellum-dependent cell motility<br>GO:0034644 - cellular response to UV<br>GO:0044010 - single-species biofilm formation<br>GO:0070301 - cellular response to hydrogen peroxide<br>GO:0006950 - response to stress
P19 H12		x		*abgR*	GO:0006355 - regulation of transcription, DNA-templated<br>GO:0006351 - transcription, DNA-templated
P21 A7			x	*wbbL*	GO:0045226 - extracellular polysaccharide biosynthetic process<br>GO:0009103 - lipopolysaccharide biosynthetic process
P21 D5		x		*ykfG*	
P21 D8		x		*yeiI*	GO:0016310 - phosphorylation
P21 D9		x		*yfbF*	GO:0006629 - lipid metabolic process<br>GO:0036108 - 4-amino-4-deoxy-alpha-L-arabinopyranosyl undecaprenyl phosphate biosynthetic process<br>GO:0046677 - response to antibiotic<br>GO:0009245 - lipid A biosynthetic process<br>GO:0009103 - lipopolysaccharide biosynthetic process
P21 D12	x			*yfhD*	GO:0009253 - peptidoglycan catabolic process<br>GO:0071555 - cell wall organization<br>GO:0000270 - peptidoglycan metabolic process<br>GO:0016998 - cell wall macromolecule catabolic process
P21 E12		x		*yfhH*	GO:0005975 - carbohydrate metabolic process<br>GO:0006355 - regulation of transcription, DNA-templated<br>GO:0006351 - transcription, DNA-templated
P21 F4		x		*ydiF*	GO:0046952 - ketone body catabolic process<br>GO:0008152 - metabolic process<br>GO:0051289 - protein homotetramerization<br>GO:0046459 - short-chain fatty acid metabolic process
P21 F9			x	*yfbB*	GO:0009234 - menaquinone biosynthetic process
P21 F12	x			*yfiC*	GO:0032259 - methylation<br>GO:0008033 - tRNA processing<br>GO:0030488 - tRNA methylation
P21 G10			x	*ypdB*	GO:0046677 - response to antibiotic<br>GO:0048870 - cell motility<br>GO:1900192 - positive regulation of single-species biofilm formation<br>GO:0006355 - regulation of transcription, DNA-templated<br>GO:0000160 - phosphorelay signal transduction system<br>GO:0006351 - transcription, DNA-templated
P23 B8		x		*gmr*	
P23 C1			x	*ygaV*	GO:0045892 - negative regulation of transcription, DNA-templated<br>GO:0006355 - regulation of transcription, DNA-templated<br>GO:0006351 - transcription, DNA-templated
P23 C3			x	*ycbL*	GO:0019243 - methylglyoxal catabolic process to D-lactate via S-lactoyl-glutathione
P23 C10			x	*yddW*	GO:0005975 - carbohydrate metabolic process
P23 F9	x			*ynbA*	GO:0008654 - phospholipid biosynthetic process
P23 G1		x		*yqaB*	GO:0016311 - dephosphorylation<br>GO:0008152 - metabolic process
P23 G8			x	*ynaI*	GO:0006810 - transport<br>GO:0055085 - transmembrane transport<br>GO:0071470 - cellular response to osmotic stress<br>GO:0006811 - ion transport
P25 D7			x	*ybaZ*	
P25 E8		x		*ybeD*	

P25 F12	x			*yccC*	GO:0000271 - polysaccharide biosynthetic process<br>GO:0045226 - extracellular polysaccharide biosynthetic process<br>GO:0016310 - phosphorylation<br>GO:0009103 - lipopolysaccharide biosynthetic process<br>GO:0038083 - peptidyl-tyrosine autophosphorylation
P25 G4		x		*thiP*	GO:0015888 - thiamine transport<br>GO:0006810 - transport
P25 H5	x			*yahA*	GO:0045893 - positive regulation of transcription, DNA-templated<br>GO:0008152 - metabolic process<br>GO:0006355 - regulation of transcription, DNA-templated
P25 H9	x			*ybhS*	GO:0006855 - drug transmembrane transport<br>GO:0055085 - transmembrane transport<br>GO:0006810 - transport
P25 H10	x			*yliG*	GO:0035600 - tRNA methylthiolation<br>GO:0043412 - macromolecule modification<br>GO:0009451 - RNA modification<br>GO:0018339 - peptidyl-L-beta-methylthioaspartic acid biosynthetic process from peptidyl-aspartic acid
P25 H12		x		*wcaJ*	GO:0000271 - polysaccharide biosynthetic process<br>GO:0009242 - colanic acid biosynthetic process<br>GO:0009103 - lipopolysaccharide biosynthetic process
P27 C4			x	*yfhL*	
P27 E1			x	*asmA*	GO:0090313 - regulation of protein targeting to membrane
P29 D6		x		*ydiT*	GO:0055114 - oxidation-reduction process
P29 G12			x	*yiaK*	GO:0008152 - metabolic process<br>GO:0055114 - oxidation-reduction process
P29 H11			x	*tar*	GO:0007165 - signal transduction<br>GO:0006935 - chemotaxis
P29 H12			x	*allD*	GO:0008152 - metabolic process<br>GO:0000256 - allantoin catabolic process<br>GO:0055114 - oxidation-reduction process<br>GO:0006144 - purine nucleobase metabolic process<br>GO:0009442 - allantoin assimilation pathway
P31 A1			x	*aceK*	GO:0006470 - protein dephosphorylation<br>GO:0016311 - dephosphorylation<br>GO:0016310 - phosphorylation<br>GO:0006006 - glucose metabolic process<br>GO:0018105 - peptidyl-serine phosphorylation<br>GO:0050790 - regulation of catalytic activity<br>GO:0006099 - tricarboxylic acid cycle<br>GO:0006097 - glyoxylate cycle
P31 B9			x	*ymgB*	GO:0071229 - cellular response to acid chemical<br>GO:0042710 - biofilm formation<br>GO:0042542 - response to hydrogen peroxide
P31 B11			x	*yddK*	
P31 D2		x		*ahpF*	GO:0045454 - cell redox homeostasis<br>GO:0000302 - response to reactive oxygen species<br>GO:0055114 - oxidation-reduction process
P31 E12		x		*yobG*	GO:0070298 - negative regulation of phosphorelay signal transduction system<br>GO:0006355 - regulation of transcription, DNA-templated<br>GO:0006351 - transcription, DNA-templated<br>GO:0044092 - negative regulation of molecular function<br>GO:0010447 - response to acidic pH<br>GO:0071286 - cellular response to magnesium ion
P31 G8	x			*elbA*	GO:0045893 - positive regulation of transcription, DNA-templated<br>GO:0009267 - cellular response to starvation<br>GO:0006950 - response to stress<br>GO:0071468 - cellular response to acidic pH<br>GO:0042177 - negative regulation of protein catabolic process<br>GO:0010350 - cellular response to magnesium starvation
P33 A3			x	*yqeB*	
P33 B1			x	*yedX*	GO:0006144 - purine nucleobase metabolic process
P33 B2			x	*ydjL*	GO:0055114 - oxidation-reduction process
P33 D10		x		*sstT*	GO:0055085 - transmembrane transport<br>GO:0003333 - amino acid transmembrane transport<br>GO:0006865 - amino acid transport<br>GO:0006810 - transport<br>GO:0015826 - threonine transport<br>GO:0006835 - dicarboxylic acid transport<br>GO:0032329 - serine transport

P33 E1			x	*yfbN*	
P33 E5		x		*yggH*	GO:0030488 - tRNA methylation<br>GO:0006400 - tRNA modification<br>GO:0036265 - RNA (guanine-N7)-methylation<br>GO:0032259 - methylation<br>GO:0008033 - tRNA processing
P33 E6		x		*yghZ*	
P33 E10		x		*ygjV*	
P33 H12	x			*nlpI*	GO:0051301 - cell division<br>GO:0007049 - cell cycle
P35 D9		x		*mdtF*	GO:0006810 - transport<br>GO:0006855 - drug transmembrane transport<br>GO:0046677 - response to antibiotic
P35 F10			x	*yiaC*	GO:0006474 - N-terminal protein amino acid acetylatior
P37 A4			x	*rmuC*	GO:0006310 - DNA recombination
P37 A6		x		*yiiR*	
P37 B8		x		*yjbI*	
P37 C1			x	*ade*	
P37 C5	x			*yihT*	GO:0061720 - 6-sulfoquinovose(1-) catabolic process to glycerone phosphate and 3-sulfolactaldehyde<br>GO:1902777 - 6-sulfoquinovose(1-) catabolic process
P37 D1	x			*yidG*	
P37 D2	x			*yieF*	GO:0055114 - oxidation-reduction process<br>GO:0006805 - xenobiotic metabolic process
P37 D3	x			*rffC*	GO:0009246 - enterobacterial common antigen biosynthetic process
P37 D4	x			*yihI*	GO:0042254 - ribosome biogenesis<br>GO:0043547 - positive regulation of GTPase activity<br>GO:0090071 - negative regulation of ribosome biogenesis
P37 D6			x	*yiiX*	
P37 D11	x			*yjeN*	
P37 E6		x		*yijF*	
P37 E7		x		*yjbD*	
P37 E8		x		*yjbM*	
P37 E11			x	*yjeO*	
P37 F3	x			*yigA*	
P37 G8		x		*yjbR*	
P37 G12		x		*yjfM*	
P37 H4	x			*ompL*	GO:0055085 - transmembrane transport<br>GO:0046323 - glucose import<br>GO:0015757 - galactose transport<br>GO:0015751 - arabinose transport<br>GO:0008643 - carbohydrate transport<br>GO:0006811 - ion transport<br>GO:0006810 - transport
P39 B6			x	*yjjK*	
P39 B10			x	*mrr*	GO:0090305 - nucleic acid phosphodiester bond hydrolysis<br>GO:0051599 - response to hydrostatic pressure<br>GO:0009307 - DNA restriction-modification system
P39 C7		x		*mfd*	GO:0006283 - transcription-coupled nucleotide-excision repair<br>GO:0006974 - cellular response to DNA damage stimulus<br>GO:0000716 - transcription-coupled nucleotide-excision repair, DNA damage recognition<br>GO:0006355 - regulation of transcription, DNA-templated<br>GO:0006281 - DNA repair
P39 D10		x		*holD*	GO:0071897 - DNA biosynthetic process<br>GO:0090305 - nucleic acid phosphodiester bond hydrolysis<br>GO:0006260 - DNA replication
P39 D12		x		*tauB*	GO:0015837 - amine transport<br>GO:0008152 - metabolic process<br>GO:0010438 - cellular response to sulfur starvation<br>GO:0015734 - taurine transport<br>GO:0006810 - transport
P39 F1		x		*ytfG*	GO:0055114 - oxidation-reduction process
P39 G1		x		*ytfJ*	
P43 A1		x		*serB*	GO:0008152 - metabolic process<br>GO:0016311 - dephosphorylation<br>GO:0008652 - cellular amino acid biosynthetic process<br>GO:0006564 - L-serine biosynthetic process

P43 A2		x		*entF*	GO:0009058 - biosynthetic process<br>GO:0008152 - metabolic process<br>GO:0043041 - amino acid activation for nonribosomal peptide biosynthetic process<br>GO:0009239 - enterobactin biosynthetic process
P43 C2	x			*entE*	GO:0019290 - siderophore biosynthetic process<br>GO:0008152 - metabolic process<br>GO:0009239 - enterobactin biosynthetic process
P43 C6			x	*menE*	GO:0051289 - protein homotetramerization<br>GO:0008152 - metabolic process<br>GO:0009234 - menaquinone biosynthetic process
P43 D2		x		*entB*	GO:0002047 - phenazine biosynthetic process<br>GO:0008152 - metabolic process<br>GO:0009239 - enterobactin biosynthetic process
P43 D11		x		*csgA*	GO:1990000 - amyloid fibril formation<br>GO:0044010 - single-species biofilm formation<br>GO:0007155 - cell adhesion
P43 D12	x			*flgG*	GO:0071978 - bacterial-type flagellum-dependent swarming motility<br>GO:0071973 - bacterial-type flagellum-dependent cell motility<br>GO:0044781 - bacterial-type flagellum organization
P43 E5	x			*cobU*	GO:0051188 - cofactor biosynthetic process<br>GO:0006974 - cellular response to DNA damage stimulus<br>GO:0006779 - porphyrin-containing compound biosynthetic process<br>GO:0016310 - phosphorylation<br>GO:0009236 - cobalamin biosynthetic process
P43 E7	x			*idi*	GO:0009240 - isopentenyl diphosphate biosynthetic process<br>GO:0050992 - dimethylallyl diphosphate biosynthetic process<br>GO:0006974 - cellular response to DNA damage stimulus<br>GO:0008299 - isoprenoid biosynthetic process
P43 E8		x		*fre*	GO:0043085 - positive regulation of catalytic activity<br>GO:0030091 - protein repair<br>GO:0006979 - response to oxidative stress<br>GO:0055072 - iron ion homeostasis<br>GO:0055114 - oxidation-reduction process<br>GO:0006811 - ion transport<br>GO:0006810 - transport
P43 E11		x		*flgN*	GO:0044781 - bacterial-type flagellum organization<br>GO:0044780 - bacterial-type flagellum assembly
P43 F3		x		*moaC*	GO:0034214 - protein hexamerization<br>GO:0032324 - molybdopterin cofactor biosynthetic process<br>GO:0006777 - Mo-molybdopterin cofactor biosynthetic process
P43 F4		x		*paaF*	GO:0006635 - fatty acid beta-oxidation<br>GO:0008152 - metabolic process<br>GO:0010124 - phenylacetate catabolic process<br>GO:0006631 - fatty acid metabolic process<br>GO:0006629 - lipid metabolic process
P43 F11		x		*flgA*	GO:0071973 - bacterial-type flagellum-dependent cell motility<br>GO:0044781 - bacterial-type flagellum organization
P43 G3	x			*moaD*	GO:0006777 - Mo-molybdopterin cofactor biosynthetic process
P43 G4	x			*paaG*	GO:0008152 - metabolic process<br>GO:0010124 - phenylacetate catabolic process
P43 G10		x		*ybgP*	GO:0061077 - chaperone-mediated protein folding<br>GO:0071555 - cell wall organization<br>GO:0043711 - pilus organization<br>GO:0006457 - protein folding
P43 G12		x		*flgK*	GO:0071973 - bacterial-type flagellum-dependent cell motility<br>GO:0044780 - bacterial-type flagellum assembly
P43 H7	x			*gshB*	GO:0006750 - glutathione biosynthetic process
P43 H8	x			*menA*	GO:0009234 - menaquinone biosynthetic process
P45 A5			x	*yraI*	GO:0061077 - chaperone-mediated protein folding<br>GO:0071555 - cell wall organization<br>GO:0006457 - protein folding<br>GO:0043711 - pilus organization
P45 A8			x	*fimA*	GO:0007155 - cell adhesion
P45 A11			x	*cheW*	GO:0051649 - establishment of localization in cell<br>GO:0007165 - signal transduction<br>GO:0006935 - chemotaxis

P45 B9			x	*ahpC*	GO:0045454 - cell redox homeostasis<br>GO:0098869 - cellular oxidant detoxification<br>GO:0033214 - iron assimilation by chelation and transport<br>GO:0006979 - response to oxidative stress<br>GO:0009970 - cellular response to sulfate starvation<br>GO:0033194 - response to hydroperoxide<br>GO:0033195 - response to alkyl hydroperoxide<br>GO:0055114 - oxidation-reduction process
P45 B10		x		*trg*	GO:0007165 - signal transduction<br>GO:0006935 - chemotaxis
P45 B11		x		*motB*	GO:0097588 - archaeal or bacterial-type flagellum-dependent cell motility<br>GO:0006935 - chemotaxis
P45 C11			x	*motA*	GO:0071978 - bacterial-type flagellum-dependent swarming motility<br>GO:0071973 - bacterial-type flagellum-dependent cell motility<br>GO:0015031 - protein transport<br>GO:0097588 - archaeal or bacterial-type flagellum-dependent cell motility<br>GO:0015992 - proton transport<br>GO:0006935 - chemotaxis<br>GO:0006811 - ion transport<br>GO:0006810 - transport
P45 D3		x		*fliL*	GO:0071978 - bacterial-type flagellum-dependent swarming motility<br>GO:0071973 - bacterial-type flagellum-dependent cell motility<br>GO:0097588 - archaeal or bacterial-type flagellum-dependent cell motility<br>GO:0006935 - chemotaxis
P45 D7		x		*rffM*	GO:0009058 - biosynthetic process<br>GO:0009246 - enterobacterial common antigen biosynthetic process
P45 D12		x		*secG*	GO:0006886 - intracellular protein transport<br>GO:0032978 - protein insertion into membrane from inner side<br>GO:0006616 - SRP-dependent cotranslational protein targeting to membrane, translocation<br>GO:0015031 - protein transport<br>GO:0006810 - transport<br>GO:0009306 - protein secretion<br>GO:0043952 - protein transport by the Sec complex<br>GO:0065002 - intracellular protein transmembrane transport
P45 F5			x	*rfaY*	GO:0009244 - lipopolysaccharide core region biosynthetic process<br>GO:0016310 - phosphorylation<br>GO:0009103 - lipopolysaccharide biosynthetic process
P45 G11			x	*hscA*	GO:0097428 - protein maturation by iron-sulfur cluster transfer<br>GO:0006457 - protein folding<br>GO:0070417 - cellular response to cold<br>GO:0016226 - iron-sulfur cluster assembly
P45 H8	x			*betB*	GO:0008152 - metabolic process<br>GO:0051289 - protein homotetramerization<br>GO:0055114 - oxidation-reduction process<br>GO:0019285 - glycine betaine biosynthetic process from choline<br>GO:0006970 - response to osmotic stress
P45 H11			x	*proX*	GO:0006972 - hyperosmotic response<br>GO:0031460 - glycine betaine transport<br>GO:0006865 - amino acid transport<br>GO:0006810 - transport
P47 A6	x			*rfbD*	GO:0019305 - dTDP-rhamnose biosynthetic process<br>GO:0055114 - oxidation-reduction process<br>GO:0045226 - extracellular polysaccharide biosynthetic process<br>GO:0009103 - lipopolysaccharide biosynthetic process<br>GO:0009243 - O antigen biosynthetic process
P47 B4			x	*putA*	GO:0045892 - negative regulation of transcription, DNA-templated<br>GO:0008152 - metabolic process<br>GO:0006560 - proline metabolic process<br>GO:0006561 - proline biosynthetic process<br>GO:0006355 - regulation of transcription, DNA-templated<br>GO:0010133 - proline catabolic process to glutamate<br>GO:0055114 - oxidation-reduction process<br>GO:0006351 - transcription, DNA-templated
P47 B7	x			*hcaB*	GO:0008152 - metabolic process<br>GO:0055114 - oxidation-reduction process<br>GO:0019439 - aromatic compound catabolic process<br>GO:0019380 - 3-phenylpropionate catabolic process
P47 B11			x	*pldB*	GO:0006629 - lipid metabolic process

P47 C11			x	*metF*	GO:0006730 - one-carbon metabolic process<br>GO:0051289 - protein homotetramerization<br>GO:0046654 - tetrahydrofolate biosynthetic process<br>GO:0006555 - methionine metabolic process<br>GO:0035999 - tetrahydrofolate interconversion<br>GO:0055114 - oxidation-reduction process<br>GO:0009086 - methionine biosynthetic process<br>GO:0008652 - cellular amino acid biosynthetic process
P47 E7	x			*cysN*	GO:0000103 - sulfate assimilation<br>GO:0070814 - hydrogen sulfide biosynthetic process<br>GO:0006790 - sulfur compound metabolic process
P47 E8	x			*gcvP*	GO:0019464 - glycine decarboxylation via glycine cleavage system<br>GO:0006546 - glycine catabolic process<br>GO:0006544 - glycine metabolic process<br>GO:0055114 - oxidation-reduction process
P47 E12		x		*treC*	GO:0008152 - metabolic process<br>GO:0005975 - carbohydrate metabolic process<br>GO:0006974 - cellular response to DNA damage stimulus<br>GO:0005993 - trehalose catabolic process
P47 H5		x		*rfbA*	GO:0045226 - extracellular polysaccharide biosynthetic process<br>GO:0009058 - biosynthetic process<br>GO:0019305 - dTDP-rhamnose biosynthetic process<br>GO:0009103 - lipopolysaccharide biosynthetic process<br>GO:0009243 - O antigen biosynthetic process
P47 H6	x			*dsdA*	GO:0070179 - D-serine biosynthetic process<br>GO:0006563 - L-serine metabolic process<br>GO:0070178 - D-serine metabolic process<br>GO:0046416 - D-amino acid metabolic process<br>GO:0006520 - cellular amino acid metabolic process<br>GO:0006974 - cellular response to DNA damage stimulus<br>GO:0036088 - D-serine catabolic process<br>GO:0051410 - detoxification of nitrogen compound
P47 H7		x		*cysI*	GO:0019419 - sulfate reduction<br>GO:0000103 - sulfate assimilation<br>GO:0070814 - hydrogen sulfide biosynthetic process<br>GO:0055114 - oxidation-reduction process<br>GO:0019344 - cysteine biosynthetic process<br>GO:0008652 - cellular amino acid biosynthetic process
P47 H8	x			*speA*	GO:0033388 - putrescine biosynthetic process from arginine<br>GO:0006596 - polyamine biosynthetic process<br>GO:0009446 - putrescine biosynthetic process<br>GO:0008295 - spermidine biosynthetic process<br>GO:0006527 - arginine catabolic process
P47 H12		x		*idnD*	GO:0055114 - oxidation-reduction process<br>GO:0019521 - D-gluconate metabolic process<br>GO:0046183 - L-idonate catabolic process
P49 B3			x	*atoB*	GO:0006635 - fatty acid beta-oxidation<br>GO:0051289 - protein homotetramerization<br>GO:0043442 - acetoacetic acid catabolic process<br>GO:0008152 - metabolic process<br>GO:0006631 - fatty acid metabolic process<br>GO:0006629 - lipid metabolic process
P49 D5		x		*gpt*	GO:0009116 - nucleoside metabolic process<br>GO:0006166 - purine ribonucleoside salvage<br>GO:0032263 - GMP salvage<br>GO:0032264 - IMP salvage<br>GO:0032265 - XMP salvage
P49 E6			x	*rnk*	GO:0032784 - regulation of DNA-templated transcription, elongation<br>GO:0065007 - biological regulation
P49 F5		x		*sbcD*	GO:0006281 - DNA repair<br>GO:0090305 - nucleic acid phosphodiester bond hydrolysis<br>GO:0006310 - DNA recombination<br>GO:0006260 - DNA replication<br>GO:0006259 - DNA metabolic process
P49 F11			x	*torA*	GO:0055114 - oxidation-reduction process<br>GO:0009060 - aerobic respiration<br>GO:0009061 - anaerobic respiration
P49 F12			x	*ydcW*	GO:0008152 - metabolic process<br>GO:0055114 - oxidation-reduction process<br>GO:0009447 - putrescine catabolic process

P49 G11			x	*grxB*	GO:0006749 - glutathione metabolic process<br>GO:0055114 - oxidation-reduction process<br>GO:0045454 - cell redox homeostasis
P49 H12	x			*narW*	GO:0051131 - chaperone-mediated protein complex assembly<br>GO:0042128 - nitrate assimilation
P51 B5	x			*napF*	GO:0055114 - oxidation-reduction process<br>GO:0006979 - response to oxidative stress
P51 D10	x			*aroB*	GO:0009073 - aromatic amino acid family biosynthetic process<br>GO:0008652 - cellular amino acid biosynthetic process<br>GO:0009423 - chorismate biosynthetic process
P51 E8		x		*yliK*	GO:0008152 - metabolic process
P51 F9		x		*uxaA*	GO:0019698 - D-galacturonate catabolic process
P51 F12		x		*grxC*	GO:0042965 - glutaredoxin biosynthetic process<br>GO:0055114 - oxidation-reduction process<br>GO:0045454 - cell redox homeostasis<br>GO:0009263 - deoxyribonucleotide biosynthetic process
P51 G10		x		*glpE*	GO:0006071 - glycerol metabolic process
P51 H1	x			*ynfG*	GO:0055114 - oxidation-reduction process
P53 B10			x	*sspB*	GO:0045732 - positive regulation of protein catabolic process
P53 C4		x		*xapB*	GO:1901642 - nucleoside transmembrane transport<br>GO:0015863 - xanthosine transport<br>GO:0015858 - nucleoside transport<br>GO:0055086 - nucleobase-containing small molecule metabolic process<br>GO:0006810 - transport
P53 C7		x		*deoC*	GO:0016052 - carbohydrate catabolic process<br>GO:0046386 - deoxyribose phosphate catabolic process<br>GO:0006974 - cellular response to DNA damage stimulus<br>GO:0009264 - deoxyribonucleotide catabolic process<br>GO:0015949 - nucleobase-containing small molecule interconversion
P53 E9	x			*relA*	GO:0042594 - response to starvation<br>GO:0008152 - metabolic process<br>GO:0015970 - guanosine tetraphosphate biosynthetic process<br>GO:0016310 - phosphorylation<br>GO:0015969 - guanosine tetraphosphate metabolic process<br>GO:0015949 - nucleobase-containing small molecule interconversion
P53 E10		x		*phoU*	GO:0055085 - transmembrane transport<br>GO:0044341 - sodium-dependent phosphate transport<br>GO:0035725 - sodium ion transmembrane transport<br>GO:0035435 - phosphate ion transmembrane transport<br>GO:0071467 - cellular response to pH<br>GO:0009405 - pathogenesis<br>GO:2000186 - negative regulation of phosphate transmembrane transport<br>GO:0071236 - cellular response to antibiotic<br>GO:0034605 - cellular response to heat<br>GO:0032413 - negative regulation of ion transmembrane transporter activity<br>GO:0030643 - cellular phosphate ion homeostasis<br>GO:0016036 - cellular response to phosphate starvation<br>GO:0010629 - negative regulation of gene expression<br>GO:0009267 - cellular response to starvation<br>GO:0001558 - regulation of cell growth<br>GO:0045936 - negative regulation of phosphate metabolic process<br>GO:0006817 - phosphate ion transport<br>GO:0006810 - transport
P53 G1	x			*fadB*	GO:0009062 - fatty acid catabolic process<br>GO:0006635 - fatty acid beta-oxidation<br>GO:0055114 - oxidation-reduction process<br>GO:0016042 - lipid catabolic process<br>GO:0008152 - metabolic process<br>GO:0006631 - fatty acid metabolic process<br>GO:0006629 - lipid metabolic process
P53 G4	x			*xseA*	GO:0090305 - nucleic acid phosphodiester bond hydrolysis<br>GO:0006308 - DNA catabolic process
P53 G5		x		*recJ*	GO:0090305 - nucleic acid phosphodiester bond hydrolysis<br>GO:0006310 - DNA recombination<br>GO:0006281 - DNA repair
P53 G7		x		*glnK*	GO:0050790 - regulation of catalytic activity<br>GO:0006808 - regulation of nitrogen utilization<br>GO:0006355 - regulation of transcription, DNA-templated<br>GO:0045848 - positive regulation of nitrogen utilization<br>GO:0006351 - transcription, DNA-templated

P53 G8	x			*feaR*	GO:0006355 - regulation of transcription, DNA-templated<br>GO:0006351 - transcription, DNA-templated<br>GO:0045893 - positive regulation of transcription, DNA-templated
P53 G10			x	*yijO*	GO:0006355 - regulation of transcription, DNA-templated<br>GO:0006351 - transcription, DNA-templated
P53 H5		x		*endA*	GO:0090305 - nucleic acid phosphodiester bond hydrolysis<br>GO:0006308 - DNA catabolic process
P53 H12		x		*pnuC*	GO:0006810 - transport<br>GO:0034258 - nicotinamide riboside transport
P55 A1		x		*modE*	GO:0055085 - transmembrane transport<br>GO:0015689 - molybdate ion transport<br>GO:0006355 - regulation of transcription, DNA-templated<br>GO:0006810 - transport<br>GO:0006351 - transcription, DNA-templated
P55 A2		x		*yliC*	GO:0006810 - transport<br>GO:0034775 - glutathione transmembrane transport
P55 A4		x		*potC*	GO:1902047 - polyamine transmembrane transport<br>GO:0006810 - transport<br>GO:0015846 - polyamine transport
P55 B2		x		*cmr*	GO:0071805 - potassium ion transmembrane transport<br>GO:0035725 - sodium ion transmembrane transport<br>GO:0030641 - regulation of cellular pH<br>GO:0006855 - drug transmembrane transport<br>GO:0055085 - transmembrane transport<br>GO:0046677 - response to antibiotic<br>GO:0006810 - transport
P55 B10		x		*mglC*	GO:0015765 - methylgalactoside transport<br>GO:0015757 - galactose transport<br>GO:0015749 - monosaccharide transport<br>GO:0006974 - cellular response to DNA damage stimulus<br>GO:0008643 - carbohydrate transport<br>GO:0006810 - transport
P55 C8		x		*ydjE*	GO:0055085 - transmembrane transport<br>GO:0006810 - transport
P55 D1		x		*ybhL*	
P55 D8		x		*manX*	GO:0034219 - carbohydrate transmembrane transport<br>GO:0061490 - glucose import into cell<br>GO:0015761 - mannose transport<br>GO:0032445 - fructose import<br>GO:0015764 - N-acetylglucosamine transport<br>GO:0008643 - carbohydrate transport<br>GO:0006810 - transport<br>GO:0016310 - phosphorylation<br>GO:0009401 - phosphoenolpyruvate-dependent sugar phosphotransferase system
P55 E7			x	*btuE*	GO:0098869 - cellular oxidant detoxification<br>GO:0006979 - response to oxidative stress<br>GO:0033194 - response to hydroperoxide<br>GO:0055114 - oxidation-reduction process
P55 F2		x		*artQ*	GO:1902765 - L-arginine import into cell<br>GO:0006865 - amino acid transport<br>GO:0006810 - transport
P55 G10		x		*glvB*	GO:0034219 - carbohydrate transmembrane transport<br>GO:0016310 - phosphorylation<br>GO:0008643 - carbohydrate transport<br>GO:0006810 - transport<br>GO:0009401 - phosphoenolpyruvate-dependent sugar phosphotransferase system
P55 H1			x	*ybiT*	GO:0008152 - metabolic process<br>GO:0009409 - response to cold<br>GO:0006810 - transport
P55 H3			x	*potD*	GO:1902047 - polyamine transmembrane transport<br>GO:0006810 - transport<br>GO:0015846 - polyamine transport
P55 H4	x			*sapD*	GO:0008152 - metabolic process<br>GO:0071805 - potassium ion transmembrane transport<br>GO:0015833 - peptide transport<br>GO:0015031 - protein transport<br>GO:0006810 - transport

P57 C1		x		*malK*	GO:0055085 - transmembrane transport<br>GO:0008152 - metabolic process<br>GO:0008643 - carbohydrate transport<br>GO:0006810 - transport<br>GO:0015768 - maltose transport<br>GO:0042956 - maltodextrin transport
P57 C5			x	*rpmF*	GO:0006412 - translation
P57 D6	x			*hyfB*	GO:0042773 - ATP synthesis coupled electron transport<br>GO:0055114 - oxidation-reduction process
P57 D7		x		*fruB*	GO:0032445 - fructose import<br>GO:0016310 - phosphorylation<br>GO:0008643 - carbohydrate transport<br>GO:0006810 - transport<br>GO:0009401 - phosphoenolpyruvate-dependent sugar phosphotransferase system
P57 D10	x			*proW*	GO:0006972 - hyperosmotic response<br>GO:0031460 - glycine betaine transport<br>GO:0015871 - choline transport<br>GO:0006865 - amino acid transport<br>GO:0006810 - transport
P57 D11	x			*yqeG*	GO:0003333 - amino acid transmembrane transport<br>GO:0006810 - transport
P57 E9		x		*focB*	GO:0015724 - formate transport<br>GO:0006810 - transport
P57 F5	x			*rpmI*	GO:0006412 - translation
P57 F9	x			*yfgO*	GO:0055085 - transmembrane transport<br>GO:0006810 - transport
P57 F11	x			*cmtA*	GO:0034219 - carbohydrate transmembrane transport<br>GO:0015797 - mannitol transport<br>GO:0016310 - phosphorylation<br>GO:0008643 - carbohydrate transport<br>GO:0006810 - transport<br>GO:0009401 - phosphoenolpyruvate-dependent sugar phosphotransferase system
P57 F12	x			*kbaY*	GO:0051289 - protein homotetramerization<br>GO:0005975 - carbohydrate metabolic process<br>GO:2001059 - D-tagatose 6-phosphate catabolic process
P57 G2		x		*treB*	GO:0043610 - regulation of carbohydrate utilization<br>GO:0034219 - carbohydrate transmembrane transport<br>GO:0015771 - trehalose transport<br>GO:0016310 - phosphorylation<br>GO:0008643 - carbohydrate transport<br>GO:0006810 - transport<br>GO:0009401 - phosphoenolpyruvate-dependent sugar phosphotransferase system
P57 G3		x		*creD*	
P57 G4	x			*aat*	GO:0030163 - protein catabolic process
P57 G5	x			*selD*	GO:0016260 - selenocysteine biosynthetic process<br>GO:0070329 - tRNA seleno-modification<br>GO:0016310 - phosphorylation
P57 G6		x		*hyfF*	GO:0006974 - cellular response to DNA damage stimulus<br>GO:0055114 - oxidation-reduction process
P57 G8		x		*ptsI*	GO:0008643 - carbohydrate transport<br>GO:0006810 - transport<br>GO:0016310 - phosphorylation<br>GO:0009401 - phosphoenolpyruvate-dependent sugar phosphotransferase system
P57 G9	x			*uraA*	GO:1903791 - uracil transmembrane transport<br>GO:0006810 - transport<br>GO:0055085 - transmembrane transport<br>GO:0015857 - uracil transport
P57 H1	x			*phnC*	GO:0015748 - organophosphate ester transport<br>GO:0008152 - metabolic process<br>GO:0015716 - organic phosphonate transport<br>GO:0006810 - transport<br>GO:0042916 - alkylphosphonate transport
P57 H4		x		*rmf*	GO:0006417 - regulation of translation<br>GO:0032055 - negative regulation of translation in response to stress<br>GO:0033554 - cellular response to stress

P57 H5	x			*rrmA*	GO:0032259 - methylation<br>GO:0006364 - rRNA processing<br>GO:0070475 - rRNA base methylation
P57 H7		x		*hisJ*	GO:1902024 - L-histidine transport<br>GO:0006865 - amino acid transport<br>GO:0006810 - transport
P57 H9	x			*yphD*	GO:0015749 - monosaccharide transport<br>GO:0006810 - transport
P57 H10		x		*gudP*	GO:0006820 - anion transport<br>GO:0055085 - transmembrane transport<br>GO:0006810 - transport
P59 B5			x	*xylF*	GO:0008643 - carbohydrate transport<br>GO:0006810 - transport<br>GO:0015752 - D-ribose transport<br>GO:0015753 - D-xylose transport
P59 B6			x	*tbpA*	GO:0071934 - thiamine transmembrane transport<br>GO:0055085 - transmembrane transport<br>GO:0015888 - thiamine transport<br>GO:0006810 - transport
P59 B7			x	*ybbW*	GO:0055085 - transmembrane transport<br>GO:0015851 - nucleobase transport<br>GO:0015720 - allantoin transport<br>GO:0006810 - transport<br>GO:0006144 - purine nucleobase metabolic process
P59 H5			x	*uhpT*	GO:0006817 - phosphate ion transport<br>GO:0055085 - transmembrane transport<br>GO:0035435 - phosphate ion transmembrane transport<br>GO:0006810 - transport<br>GO:0008643 - carbohydrate transport<br>GO:0015712 - hexose phosphate transport<br>GO:0015760 - glucose-6-phosphate transport
P61 A4			x	*panF*	GO:0071436 - sodium ion export<br>GO:0055085 - transmembrane transport<br>GO:0015887 - pantothenate transmembrane transport<br>GO:0006814 - sodium ion transport<br>GO:0006811 - ion transport<br>GO:0006810 - transport
P61 A8			x	*rusA*	GO:0090305 - nucleic acid phosphodiester bond hydrolysis<br>GO:0006974 - cellular response to DNA damage stimulus<br>GO:0006310 - DNA recombination<br>GO:0006281 - DNA repair
P61 A10	x			*ampE*	
P61 C2		x		*yggL*	
P61 C5		x		*prmA*	GO:0032259 - methylation<br>GO:0006479 - protein methylation<br>GO:0018023 - peptidyl-lysine trimethylation<br>GO:0018012 - N-terminal peptidyl-alanine trimethylation
P61 C6		x		*dtd*	GO:0006450 - regulation of translational fidelity<br>GO:0009408 - response to heat<br>GO:0019478 - D-amino acid catabolic process<br>GO:0006399 - tRNA metabolic process
P61 C9		x		*rho*	GO:0006355 - regulation of transcription, DNA-templated<br>GO:0006353 - DNA-templated transcription, termination<br>GO:0006351 - transcription, DNA-templated
P61 D8		x		*dgsA*	GO:0005975 - carbohydrate metabolic process<br>GO:0006355 - regulation of transcription, DNA-templated<br>GO:0006351 - transcription, DNA-templated
P61 D12		x		*cspD*	GO:0006950 - response to stress<br>GO:0006355 - regulation of transcription, DNA-templated<br>GO:0042594 - response to starvation<br>GO:0008156 - negative regulation of DNA replication
P61 E1		x		*ygcM*	GO:0008616 - queuosine biosynthetic process
P61 E4		x		*frlA*	GO:1902475 - L-alpha-amino acid transmembrane transport<br>GO:0015807 - L-amino acid transport<br>GO:0003333 - amino acid transmembrane transport<br>GO:0030393 - fructoselysine metabolic process<br>GO:0006865 - amino acid transport<br>GO:0006810 - transport
P61 E8		x		*yegW*	GO:0006355 - regulation of transcription, DNA-templated<br>GO:0006351 - transcription, DNA-templated
P61 E11		x		*borD*	GO:0071286 - cellular response to magnesium ion

P61 F3	x			*elbB*	GO:0045828 - positive regulation of isoprenoid metabolic process<br>GO:0008299 - isoprenoid biosynthetic process
P61 F6		x		*rplK*	GO:0000027 - ribosomal large subunit assembly<br>GO:0006412 - translation<br>GO:0015968 - stringent response<br>GO:0006415 - translational termination
P61 F10		x		*ampG*	GO:0006810 - transport<br>GO:0055085 - transmembrane transport<br>GO:0015835 - peptidoglycan transport<br>GO:0000270 - peptidoglycan metabolic process
P61 F11		x		*nfrB*	GO:0006810 - transport
P61 G10		x		*ybaA*	
P61 H8		x		*cpdA*	GO:0042545 - cell wall modification<br>GO:0008152 - metabolic process
P63 B1		x		*ymfG*	GO:0006310 - DNA recombination
P63 C7		x		*yiiT*	GO:0006950 - response to stress
P63 D5		x		*emrA*	GO:0055085 - transmembrane transport<br>GO:0006855 - drug transmembrane transport<br>GO:0046677 - response to antibiotic<br>GO:0006810 - transport
P63 E6		x		*acpT*	GO:0019878 - lysine biosynthetic process via aminoadipic acid<br>GO:0018070 - peptidyl-serine phosphopantetheinylation<br>GO:0006633 - fatty acid biosynthetic process
P63 G12			x	*ulaC*	GO:0034219 - carbohydrate transmembrane transport<br>GO:0016310 - phosphorylation<br>GO:0006810 - transport<br>GO:0015882 - L-ascorbic acid transport<br>GO:0009401 - phosphoenolpyruvate-dependent sugar phosphotransferase system
P63 H9		x		*hemN*	GO:0006782 - protoporphyrinogen IX biosynthetic process<br>GO:0006779 - porphyrin-containing compound biosynthetic process<br>GO:0019353 - protoporphyrinogen IX biosynthetic process from glutamate<br>GO:0055114 - oxidation-reduction process
P65 E9			x	*yhiL*	
P65 F8		x		*gltB*	GO:0019676 - ammonia assimilation cycle<br>GO:0008652 - cellular amino acid biosynthetic process<br>GO:0008152 - metabolic process<br>GO:0097054 - L-glutamate biosynthetic process<br>GO:0006807 - nitrogen compound metabolic process<br>GO:0055114 - oxidation-reduction process<br>GO:0006541 - glutamine metabolic process<br>GO:0006537 - glutamate biosynthetic process
P65 F11		x		*dsbA*	
P65 F12		x		*ytfA*	
P65 G6			x	*fucK*	GO:0046835 - carbohydrate phosphorylation<br>GO:0019317 - fucose catabolic process<br>GO:0006004 - fucose metabolic process<br>GO:0005975 - carbohydrate metabolic process<br>GO:0016310 - phosphorylation<br>GO:0042355 - L-fucose catabolic process<br>GO:0019571 - D-arabinose catabolic process
P65 G7			x	*tdcC*	GO:0015992 - proton transport<br>GO:0032329 - serine transport<br>GO:0006810 - transport<br>GO:0006865 - amino acid transport<br>GO:0003333 - amino acid transmembrane transport<br>GO:0015825 - L-serine transport<br>GO:0015826 - threonine transport
P67 A1		x		*yjgX*	GO:0009244 - lipopolysaccharide core region biosynthetic process<br>GO:0008152 - metabolic process
P67 A8		x		*mgsA*	GO:0019242 - methylglyoxal biosynthetic process
P67 E9		x		*sieB*	
P69 B3			x	*menD*	GO:0009234 - menaquinone biosynthetic process
P69 B5	x			*amiC*	GO:0051301 - cell division<br>GO:0071555 - cell wall organization<br>GO:0043093 - FtsZ-dependent cytokinesis<br>GO:0009253 - peptidoglycan catabolic process
P69 B8	x			*yghX*	GO:0008152 - metabolic process

P69 B12			x	*kdgT*	GO:0015992 - proton transport<br>GO:0008643 - carbohydrate transport<br>GO:0006810 - transport<br>GO:0046411 - 2-keto-3-deoxygluconate transport<br>GO:0035429 - gluconate transmembrane transport
P69 C3		x		*torI*	GO:0006355 - regulation of transcription, DNA-templated<br>GO:0006351 - transcription, DNA-templated<br>GO:2000143 - negative regulation of DNA-templated transcription, initiation<br>GO:0044349 - DNA excision<br>GO:0006310 - DNA recombination
P69 C6	x			*ygfU*	GO:1903825 - organic acid transmembrane transport<br>GO:0006811 - ion transport<br>GO:0006810 - transport<br>GO:0015992 - proton transport<br>GO:0055085 - transmembrane transport<br>GO:0015747 - urate transport<br>GO:0042906 - xanthine transport
P69 C8		x		*yghY*	GO:0008152 - metabolic process
P69 D1		x		*yoeF*	GO:0006576 - cellular biogenic amine metabolic process
P69 F1		x		*yoeB*	GO:0098795 - mRNA cleavage involved in gene silencing<br>GO:1903507 - negative regulation of nucleic acid-templated transcription<br>GO:0090502 - RNA phosphodiester bond hydrolysis, endonucleolytic<br>GO:0006402 - mRNA catabolic process<br>GO:0044010 - single-species biofilm formation<br>GO:0045947 - negative regulation of translational initiation<br>GO:0006401 - RNA catabolic process<br>GO:0006355 - regulation of transcription, DNA-templated<br>GO:0006351 - transcription, DNA-templated
P69 F2		x		*bcr*	GO:0015833 - peptide transport<br>GO:0015031 - protein transport<br>GO:0015893 - drug transport<br>GO:0035442 - dipeptide transmembrane transport<br>GO:0006855 - drug transmembrane transport<br>GO:0055085 - transmembrane transport<br>GO:0046677 - response to antibiotic<br>GO:0006810 - transport
P69 F5			x	*xdhA*	GO:0009115 - xanthine catabolic process<br>GO:0009114 - hypoxanthine catabolic process<br>GO:0055114 - oxidation-reduction process<br>GO:0006166 - purine ribonucleoside salvage<br>GO:0006144 - purine nucleobase metabolic process
P69 G10	x			*yhdJ*	GO:0032775 - DNA methylation on adenine<br>GO:0032259 - methylation<br>GO:0006306 - DNA methylation
P69 H7		x		*hybF*	GO:0006464 - cellular protein modification process<br>GO:0051604 - protein maturation
P69 H8	x			*yqiH*	GO:0061077 - chaperone-mediated protein folding<br>GO:0071555 - cell wall organization<br>GO:0043711 - pilus organization<br>GO:0006974 - cellular response to DNA damage stimulus
P69 H10		x		*yhdX*	GO:0006865 - amino acid transport<br>GO:0006810 - transport
P69 H11		x		*ptsA*	GO:0016310 - phosphorylation<br>GO:0009401 - phosphoenolpyruvate-dependent sugar phosphotransferase system<br>GO:0008643 - carbohydrate transport<br>GO:0006810 - transport
P71 A4			x	*yicO*	GO:1904823 - purine nucleobase transmembrane transport<br>GO:0006863 - purine nucleobase transport<br>GO:0015853 - adenine transport<br>GO:0055085 - transmembrane transport<br>GO:0006810 - transport
P71 A8			x	*yhhT*	GO:0055085 - transmembrane transport
P71 B9		x		*yhfY*	GO:0006355 - regulation of transcription, DNA-templated
P71 C5			x	*yiaB*	GO:0006974 - cellular response to DNA damage stimulus
P71 F10			x	*ubiC*	GO:0042866 - pyruvate biosynthetic process<br>GO:0006744 - ubiquinone biosynthetic process
P73 A11			x	*yadD*	
P73 B4			x	*fimI*	GO:0007155 - cell adhesion<br>GO:0009297 - pilus assembly
P73 D7	x			*ygiQ*	GO:0008152 - metabolic process

P73 D8	x			*rhtB*	GO:0006865 - amino acid transport<br>GO:0006810 - transport<br>GO:0042968 - homoserine transport
P75 B4		x		*ybgQ*	GO:0055085 - transmembrane transport<br>GO:0009297 - pilus assembly<br>GO:0006810 - transport
P75 B10			x	*yciO*	
P75 C1	x			*yahM*	
P75 C4		x		*ybhT*	GO:0006855 - drug transmembrane transport<br>GO:0046677 - response to antibiotic<br>GO:0006810 - transport<br>GO:0036460 - cellular response to cell envelope stress
P75 C9			x	*ycgH*	
P75 E8		x		*ymfE*	
P75 E9	x			*ycgN*	
P75 E12	x			*ydcO*	GO:0042919 - benzoate transport<br>GO:0055085 - transmembrane transport
P75 G4		x		*ybiO*	GO:0006811 - ion transport<br>GO:0006810 - transport<br>GO:0055085 - transmembrane transport<br>GO:0071470 - cellular response to osmotic stress
P75 G8		x		*ymfT*	
P77 B10			x	*yfbE*	GO:0046493 - lipid A metabolic process<br>GO:0006629 - lipid metabolic process<br>GO:0046677 - response to antibiotic<br>GO:0009103 - lipopolysaccharide biosynthetic process<br>GO:0009245 - lipid A biosynthetic process
P77 E8		x		*yohN*	GO:1901652 - response to peptide<br>GO:0006974 - cellular response to DNA damage stimulus<br>GO:0010045 - response to nickel cation<br>GO:0032025 - response to cobalt ion
P79 B5		x		*nadR*	GO:0045892 - negative regulation of transcription, DNA-templated<br>GO:0009058 - biosynthetic process<br>GO:0006810 - transport<br>GO:0009435 - NAD biosynthetic process<br>GO:0016310 - phosphorylation<br>GO:0008152 - metabolic process<br>GO:0006355 - regulation of transcription, DNA-templated<br>GO:0019363 - pyridine nucleotide biosynthetic process<br>GO:0006351 - transcription, DNA-templated
P79 C9		x		*modA*	GO:0015689 - molybdate ion transport<br>GO:0006810 - transport
P79 D1			x	*yqaC*	GO:0005975 - carbohydrate metabolic process
P79 D6		x		*ybfO*	GO:0097264 - self proteolysis
P79 D9	x			*malT*	GO:0045913 - positive regulation of carbohydrate metabolic process<br>GO:0045893 - positive regulation of transcription, DNA-templated<br>GO:0006355 - regulation of transcription, DNA-templated<br>GO:0006351 - transcription, DNA-templated<br>GO:0005975 - carbohydrate metabolic process
P79 E5		x		*ybhR*	GO:0006855 - drug transmembrane transport<br>GO:0055085 - transmembrane transport<br>GO:0006974 - cellular response to DNA damage stimulus<br>GO:0006810 - transport
P79 F9		x		*ydhB*	GO:0006355 - regulation of transcription, DNA-templated<br>GO:0006351 - transcription, DNA-templated
P79 F12		x		*mutS*	GO:0006281 - DNA repair<br>GO:0006974 - cellular response to DNA damage stimulus<br>GO:0000018 - regulation of DNA recombination<br>GO:0006298 - mismatch repair
P79 G6		x		*rhsD*	GO:0097264 - self proteolysis<br>GO:0010438 - cellular response to sulfur starvation
P79 G9	x			*rtcB*	GO:0006974 - cellular response to DNA damage stimulus<br>GO:0006281 - DNA repair<br>GO:0006266 - DNA ligation<br>GO:0006396 - RNA processing<br>GO:0042245 - RNA repair
P79 G10	x			*rspB*	GO:0055114 - oxidation-reduction process

P79 G12		x		*mutH*	GO:0090305 - nucleic acid phosphodiester bond hydrolysis<br>GO:0006974 - cellular response to DNA damage stimulus<br>GO:0006281 - DNA repair<br>GO:0006304 - DNA modification<br>GO:0000018 - regulation of DNA recombination<br>GO:0006298 - mismatch repair
P81 A1			x	*mutY*	GO:0008152 - metabolic process<br>GO:0006974 - cellular response to DNA damage stimulus<br>GO:0006281 - DNA repair<br>GO:0006284 - base-excision repair
P81 A2			x	*gidB*	GO:0070476 - rRNA (guanine-N7)-methylation<br>GO:0032259 - methylation<br>GO:0006364 - rRNA processing<br>GO:0070475 - rRNA base methylation
P81 F5			x	*ycfJ*	GO:1900190 - regulation of single-species biofilm formation
P83 A6			x	*yhgG*	
P83 A7			x	*hemY*	GO:0042168 - heme metabolic process<br>GO:0006779 - porphyrin-containing compound biosynthetic process
P83 A11			x	*gabT*	GO:0042450 - arginine biosynthetic process via ornithine<br>GO:0009448 - gamma-aminobutyric acid metabolic process<br>GO:0009450 - gamma-aminobutyric acid catabolic process
P83 B1			x	*yfeN*	
P83 B6			x	*yzgL*	
P83 H1		x		*ydfE*	
P83 H5	x			*yhfT*	
P83 H6	x			*yibI*	
P83 H9	x			*ppdA*	
P83 H12	x			*glf*	GO:0009103 - lipopolysaccharide biosynthetic process<br>GO:0009243 - O antigen biosynthetic process
P85 A8		x		*alsA*	GO:0015754 - allose transport<br>GO:0015749 - monosaccharide transport<br>GO:0008152 - metabolic process<br>GO:0008643 - carbohydrate transport<br>GO:0006810 - transport
P85 C6			x	*yliD*	GO:0006810 - transport<br>GO:0034775 - glutathione transmembrane transport
P85 C9			x	*ssuE*	GO:0051289 - protein homotetramerization<br>GO:0006974 - cellular response to DNA damage stimulus<br>GO:0055114 - oxidation-reduction process<br>GO:0046306 - alkanesulfonate catabolic process<br>GO:0009970 - cellular response to sulfate starvation
P85 C10			x	*mhpD*	GO:0008152 - metabolic process<br>GO:0019380 - 3-phenylpropionate catabolic process<br>GO:0019439 - aromatic compound catabolic process
P85 D2			x	*fucI*	GO:0019317 - fucose catabolic process<br>GO:0006004 - fucose metabolic process<br>GO:0005996 - monosaccharide metabolic process<br>GO:0005975 - carbohydrate metabolic process<br>GO:0019571 - D-arabinose catabolic process<br>GO:0042355 - L-fucose catabolic process
P85 E2		x		*fucU*	GO:0042354 - L-fucose metabolic process<br>GO:0005996 - monosaccharide metabolic process<br>GO:0006004 - fucose metabolic process<br>GO:0005975 - carbohydrate metabolic process
P85 F6		x		*yecC*	GO:0015830 - diaminopimelate transport<br>GO:1903712 - cysteine transmembrane transport<br>GO:0008152 - metabolic process<br>GO:0003333 - amino acid transmembrane transport<br>GO:0015811 - L-cystine transport<br>GO:0006865 - amino acid transport<br>GO:0006810 - transport
P85 F9		x		*alpA*	GO:0006355 - regulation of transcription, DNA-templated<br>GO:0006351 - transcription, DNA-templated<br>GO:0042710 - biofilm formation<br>GO:0045893 - positive regulation of transcription, DNA-templated
P85 H1		x		*hypE*	GO:0046892 - peptidyl-S-carbamoyl-L-cysteine dehydration<br>GO:0051604 - protein maturation

P85 H5	x			*acrA*	GO:0015721 - bile acid and bile salt transport<br>GO:0055085 - transmembrane transport<br>GO:0006810 - transport<br>GO:0006855 - drug transmembrane transport<br>GO:0046677 - response to antibiotic
P87 B2		x		*yhiM*	GO:0071229 - cellular response to acid chemical
P87 B5		x		*ydbJ*	
P87 B7		x		*pcnB*	GO:0043631 - RNA polyadenylation<br>GO:0006396 - RNA processing<br>GO:0006397 - mRNA processing<br>GO:0006378 - mRNA polyadenylation<br>GO:0006351 - transcription, DNA-templated<br>GO:0006276 - plasmid maintenance<br>GO:0009451 - RNA modification
P87 C5	x			*yncH*	
P87 C9	x			*asr*	GO:0010447 - response to acidic pH<br>GO:0001101 - response to acid chemical<br>GO:0010043 - response to zinc ion
P87 D4	x			*hokE*	GO:0006974 - cellular response to DNA damage stimulus
P87 D11		x		*ygcS*	GO:0015850 - organic hydroxy compound transport<br>GO:0055085 - transmembrane transport<br>GO:0006810 - transport
P87 E2		x		*yjdC*	GO:0006355 - regulation of transcription, DNA-templated<br>GO:0006351 - transcription, DNA-templated
P87 E3		x		*yjjM*	GO:0006351 - transcription, DNA-templated<br>GO:0019584 - galactonate catabolic process<br>GO:0006355 - regulation of transcription, DNA-templated
P87 E6		x		*ygdI*	
P87 E8		x		*ycfS*	GO:0071555 - cell wall organization<br>GO:0006508 - proteolysis<br>GO:0009252 - peptidoglycan biosynthetic process<br>GO:0008360 - regulation of cell shape<br>GO:0043164 - Gram-negative-bacterium-type cell wall biogenesis<br>GO:0018104 - peptidoglycan-protein cross-linking
P87 E10		x		*yohG*	GO:0006810 - transport<br>GO:0046677 - response to antibiotic
P87 E11		x		*ygeO*	GO:0009405 - pathogenesis
P87 F8		x		*emtA*	GO:0071555 - cell wall organization<br>GO:0000270 - peptidoglycan metabolic process<br>GO:0016998 - cell wall macromolecule catabolic process
P87 G1		x		*yieP*	GO:0006355 - regulation of transcription, DNA-templated<br>GO:0006351 - transcription, DNA-templated
P87 G3	x			*tnaB*	GO:0006865 - amino acid transport<br>GO:0006810 - transport<br>GO:0015801 - aromatic amino acid transport<br>GO:0003333 - amino acid transmembrane transport<br>GO:0006569 - tryptophan catabolic process
P87 G4	x			*ymfP*	
P87 G8	x			*abgT*	GO:1902604 - p-aminobenzoyl-glutamate transmembrane transpor<br>GO:0006865 - amino acid transport<br>GO:0006810 - transport<br>GO:0015814 - p-aminobenzoyl-glutamate transport
P87 H5		x		*nuoC*	GO:0055114 - oxidation-reduction process<br>GO:0008152 - metabolic process<br>GO:0006810 - transport
P87 H9		x		*yedQ*	GO:1902201 - negative regulation of bacterial-type flagellum-dependent cell motility<br>GO:0030244 - cellulose biosynthetic process
P89 B4		x		*ybbN*	GO:0055114 - oxidation-reduction process<br>GO:0061077 - chaperone-mediated protein folding<br>GO:0045454 - cell redox homeostasis<br>GO:0006662 - glycerol ether metabolic process
P89 B8		x		*yibK*	GO:0032259 - methylation<br>GO:0030488 - tRNA methylation<br>GO:0008033 - tRNA processing<br>GO:0006396 - RNA processing<br>GO:0001510 - RNA methylation<br>GO:0002131 - wobble position cytosine ribose methylation<br>GO:0002132 - wobble position uridine ribose methylation
P89 E8		x		*yaaY*	GO:0006974 - cellular response to DNA damage stimulus

P89 E9		x		*ygcW*	GO:0008152 - metabolic process<br>GO:0006974 - cellular response to DNA damage stimulus<br>GO:0055114 - oxidation-reduction process
P89 E12	x			*yfeH*	
P89 G5	x			*rpsT*	GO:0043086 - negative regulation of catalytic activity<br>GO:0006412 - translation
P89 G6	x			*ybiM*	GO:0009242 - colanic acid biosynthetic process
P89 G9		x		*rpsU*	GO:0006412 - translation
P89 G10		x		*yfeS*	
P89 H5		x		*marC*	
P89 H6		x		*dmsD*	GO:0061077 - chaperone-mediated protein folding
P89 H10		x		*baeR*	GO:0006355 - regulation of transcription, DNA-templated<br>GO:0006351 - transcription, DNA-templated<br>GO:2001023 - regulation of response to drug<br>GO:2000144 - positive regulation of DNA-templated transcription, initiation<br>GO:0000160 - phosphorelay signal transduction system
P89 H11		x		*yeeP*	GO:0000027 - ribosomal large subunit assembly
P91 F1		x		*ydfK*	
P91 H2			x	*ydjR*	
P93 D1	x			*ubiX*	GO:0051188 - cofactor biosynthetic process<br>GO:0009108 - coenzyme biosynthetic process<br>GO:0006744 - ubiquinone biosynthetic process
P93 D2		x		*atpB*	GO:0015986 - ATP synthesis coupled proton transport<br>GO:0042777 - plasma membrane ATP synthesis coupled proton transport<br>GO:0015992 - proton transport<br>GO:0006811 - ion transport<br>GO:0006810 - transport<br>GO:0006754 - ATP biosynthetic process
P93 G1	x			*phnN*	GO:0046835 - carbohydrate phosphorylation<br>GO:0019634 - organic phosphonate metabolic process<br>GO:0006015 - 5-phosphoribose 1-diphosphate biosynthetic process<br>GO:0009435 - NAD biosynthetic process
P95 A1		x		*ompR*	GO:0045893 - positive regulation of transcription, DNA-templated<br>GO:0006355 - regulation of transcription, DNA-templated<br>GO:0000160 - phosphorelay signal transduction system<br>GO:0006351 - transcription, DNA-templated
P95 A2		x		*secB*	GO:0051262 - protein tetramerization<br>GO:0043952 - protein transport by the Sec complex<br>GO:0015031 - protein transport<br>GO:0006605 - protein targeting<br>GO:0008104 - protein localization<br>GO:0061077 - chaperone-mediated protein folding<br>GO:0072321 - chaperone-mediated protein transport<br>GO:0006810 - transport<br>GO:0006457 - protein folding
P95 A5			x	*rbfA*	GO:0042254 - ribosome biogenesis<br>GO:0006364 - rRNA processing<br>GO:0006974 - cellular response to DNA damage stimulus<br>GO:0009409 - response to cold<br>GO:0030490 - maturation of SSU-rRNA
P95 A6			x	*ybeY*	GO:0006508 - proteolysis<br>GO:0042254 - ribosome biogenesis<br>GO:0031564 - transcription antitermination<br>GO:0000478 - endonucleolytic cleavage involved in rRNA processing<br>GO:0030490 - maturation of SSU-rRNA<br>GO:0006364 - rRNA processing<br>GO:0009408 - response to heat<br>GO:0042274 - ribosomal small subunit biogenesis<br>GO:0006412 - translation
P95 C3			x	*rpmJ*	GO:0006412 - translation
P95 D2		x		*rnhA*	GO:0090305 - nucleic acid phosphodiester bond hydrolysis<br>GO:0090502 - RNA phosphodiester bond hydrolysis, endonucleolytic<br>GO:0006401 - RNA catabolic process<br>GO:0043137 - DNA replication, removal of RNA primer
P95 D3		x		*nusB*	GO:0006355 - regulation of transcription, DNA-templated<br>GO:0006353 - DNA-templated transcription, termination<br>GO:0006351 - transcription, DNA-templated

P95 D4			x	*envC*	GO:0051345 - positive regulation of hydrolase activity<br>GO:0001896 - autolysis<br>GO:0009273 - peptidoglycan-based cell wall biogenesis<br>GO:0051301 - cell division<br>GO:0007049 - cell cycle<br>GO:0000920 - cell separation after cytokinesis<br>GO:0042493 - response to drug
P95 F1	x			*yfgA*	
P95 F2		x		*fdx*	GO:0055114 - oxidation-reduction process<br>GO:0016226 - iron-sulfur cluster assembly
P95 F4		x		*parC*	GO:0006268 - DNA unwinding involved in DNA replication<br>GO:0006259 - DNA metabolic process<br>GO:0030541 - plasmid partitioning<br>GO:0007062 - sister chromatid cohesion<br>GO:0007059 - chromosome segregation<br>GO:0006265 - DNA topological change
P95 G1		x		*dnaK*	GO:1903506 - regulation of nucleic acid-templated transcription<br>GO:0006461 - protein complex assembly<br>GO:0043241 - protein complex disassembly<br>GO:0051085 - chaperone mediated protein folding requiring cofactor<br>GO:0070389 - chaperone cofactor-dependent protein refolding<br>GO:0034620 - cellular response to unfolded protein<br>GO:0006457 - protein folding<br>GO:0043335 - protein unfolding<br>GO:0009408 - response to heat<br>GO:0006260 - DNA replication
P95 G2		x		*rluD*	GO:0009451 - RNA modification<br>GO:0006364 - rRNA processing<br>GO:0001522 - pseudouridine synthesis<br>GO:0000455 - enzyme-directed rRNA pseudouridine synthesis<br>GO:0000027 - ribosomal large subunit assembly
P95 G3		x		*rfaE*	GO:0046835 - carbohydrate phosphorylation<br>GO:0009058 - biosynthetic process<br>GO:0097171 - ADP-L-glycero-beta-D-manno-heptose biosynthetic process<br>GO:0009244 - lipopolysaccharide core region biosynthetic process<br>GO:0016310 - phosphorylation<br>GO:0008152 - metabolic process<br>GO:0009103 - lipopolysaccharide biosynthetic process<br>GO:0005975 - carbohydrate metabolic process
